# Microwave technologies for biomedical diagnosis and therapy: advances, challenges, and perspectives

**DOI:** 10.1007/s00604-025-07826-2

**Published:** 2026-03-04

**Authors:** Sebastian Montoya-Villada, Erick Reyes-Vera, Jahir Orozco

**Affiliations:** 1 Department of Systems, Institución Universitaria ITM, Medellín, Colombia; 2 Department of Electronics and Telecommunications, Institución Universitaria ITM, Medellin, Colombia; 3 Max Planck Tandem Group in Nanobioengineering, Institute of Chemistry, Faculty of Natural and Exact Sciences, University of Antioquia, Complejo Ruta N, Medellin, 050010 Colombia

## Abstract

Microwave technologies have emerged as powerful tools for biomedical diagnosis and therapy due to their non-ionizing nature, sensitivity to dielectric changes, and compatibility with compact, low-cost systems. This review provides a unified perspective that integrates the biophysical foundations of dielectric characterization with advances in biosensing, imaging, and therapeutic microwave platforms. A systematic examination of the primary material characterization techniques—non-resonant, transmission-line resonant, and metamaterial-based methods—highlights how their frequency-dependent interactions with biological media underpin molecular, cellular, and tissue-level sensing. We synthesize recent progress in microstrip and resonant biosensors for detecting biochemical, metabolic, oncological, and pathogenic biomarkers, alongside developments in microwave imaging for functional tissue assessment and energy-based therapies such as hyperthermia and ablation. This integrated framework clarifies how the methodological diversity of microwave systems supports increasingly specialized biomedical applications. Despite their promise, current microwave technologies face several barriers to clinical translation, including variability in dielectric models, calibration and reproducibility challenges, spatial-resolution constraints, and limited in vivo validation. Addressing these limitations requires advancing beyond traditional architectures. Accordingly, this review also consolidates emerging trends in microfluidics, wearable and implantable platforms, artificial intelligence (AI)-driven signal interpretation, and multimodal integration, illustrating how these capabilities enhance sensing performance, enable adaptive therapy monitoring, and advance microwave systems toward personalized theranostic applications. By articulating the interplay between methods, capabilities, and unresolved challenges, a comprehensive and timely roadmap is offered that distinguishes microwave technologies from conventional diagnostic modalities and underscores their unique potential in next-generation biomedical engineering.

**Graphical Abstract:**

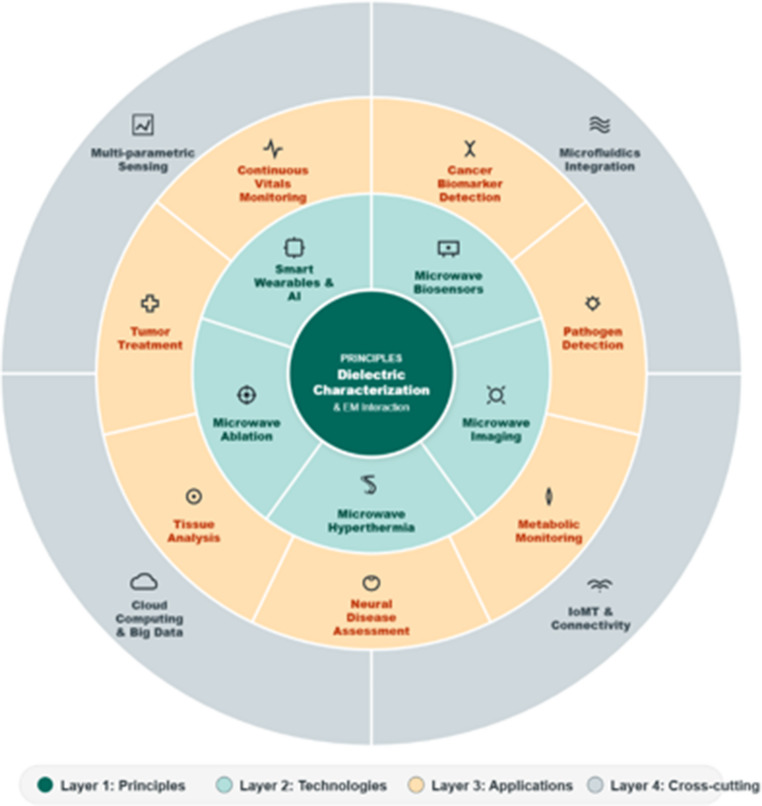

## Introduction

The frequency and incidence of chronic, degenerative, and infectious diseases are on the rise in modern healthcare systems, putting conventional diagnosis and treatment methods to the test and demanding early, accurate, and efficient solutions. Biomedical sciences are progressively emphasizing non-invasive procedures, real-time measurements, and downsizing diagnostic tools. The World Health Organization (WHO) reports that noncommunicable diseases like cancer, cardiovascular problems, and diabetes kill over 40 million people each year, accounting for almost 75% of total global mortality [1]. These conditions now eclipse many infectious diseases in terms of health, economic, and social impact [2–4]. To address these challenges, it is essential to develop medical strategies that can detect such conditions not only rapidly but also with high specificity, accuracy, and efficiency. Although conventional technologies—such as biochemical assays, imaging techniques, and molecular platforms like polymerase chain reaction (PCR), enzyme-linked immunosorbent assay (ELISA), and mass spectrometry—remain powerful tools, they often demand expensive equipment, complex sample preparation, and specialized personnel [5–8]. In response, recent technological advances have led to the emergence of innovative approaches based on biosensors, advanced imaging systems, molecular diagnostics, and point-of-care devices, offering promising diagnostic and therapeutic solutions even in resource-limited settings [9, 10]. Within this context, analytical technologies capable of probing intrinsic physical properties of biological tissues, rather than relying exclusively on molecular labeling, are gaining increasing relevance for scalable and cost-effective biomedical diagnostics.

One of the most promising frontiers in biomedical engineering emerges from the convergence of electromagnetic transduction and the life sciences, particularly with the use of microwave technologies operating in the 300 MHz to 300 GHz range. These methods exploit interactions with biological tissues and fluids through their dielectric properties—specifically, relative permittivity and conductivity—enabling label-free, non-invasive detection and monitoring of pathological processes [11]. When a disease alters the molecular composition or structural organization of tissues, it inevitably modifies their dielectric response. Such changes can be detected through microwave interrogation, allowing for the identification and real-time tracking of various conditions without the need for contrast agents or invasive sampling [12–14].

Beyond biochemical and bioclinical sensing, microwave technologies support a wide spectrum of biomedical applications that exploit their sensitivity to volumetric dielectric properties and their ability to penetrate biological tissues. These include non-invasive tissue characterization, hydration and edema assessment, continuous physiological monitoring using wearable or implantable antennas, dielectric evaluation of neural tissues for cognitive or neurodegenerative disease assessment, and real-time tracking of organ-level physiological states. In parallel, controlled microwave energy delivery has enabled therapeutic innovations such as hyperthermia, thermal ablation, neuromodulation, wound-healing stimulation, and triggered drug release using thermo-responsive systems. These diverse diagnostic and therapeutic roles demonstrate that microwave technologies constitute a multiscale biomedical platform that extends well beyond purely biochemical or biopharmaceutical contexts and motivate the integrative perspective developed throughout this review.

Microwave-based devices have shown great adaptability in both diagnostic and therapeutic domains, thanks to their ability to interact sensitively with the dielectric characteristics of biological media. Electromagnetic biosensors, which operate between 1 and 40 GHz, can detect a variety of biological targets, including proteins, metabolites, antibodies, and entire cells [11, 15, 16]. These devices work by detecting small changes in resonance frequency, characteristic impedance, or scattering parameters (S-parameters) that are affected by the presence and concentration of specific analytes [17–19]. Their value has been investigated in various biomedical applications, including cancer detection, metabolic monitoring, and infectious disease detection, where high sensitivity, label-free operation, and miniaturization provide significant benefits. Notably, different sensor topologies, such as split-ring resonators, stepped-impedance resonators, interdigital capacitive structures, and substrate-integrated waveguides, have shown remarkable improvements in sensitivity, selectivity, and compactness [19–24]. Furthermore, broadband coplanar waveguide sensors have demonstrated the ability to distinguish cellular phenotypes based on discrete dielectric signatures, underscoring the potential of microwave sensing for real-time, non-invasive cellular investigation. More recently, the integration of these platforms with advanced readout electronics and AI-based methods has further expanded their applicability, paving the way for accurate biomarker detection and personalized diagnostic strategies [25].

Microwave technologies now play an increasingly broad role across multiple biomedical domains, including real-time tissue monitoring, physiological sensing, neural stimulation, controlled drug delivery, and energy-based therapies [26–29]. Their ability to interrogate tissues through intrinsic dielectric signatures enables applications ranging from cancer staging and edema assessment to continuous monitoring of hydration, glucose levels, and cellular viability [29, 30]. Recent progress in wearable and implantable microwave antennas has expanded their use in neuromodulation, wound healing, and regenerative medicine, while high-power applicators support minimally invasive hyperthermia and ablation procedures [31–33].

In contrast to conventional diagnostic technologies such as biochemical assays, optical spectroscopy, and radiological imaging, microwave-based methods offer a unique combination of label-free operation, low-cost hardware, real-time response, and compatibility with miniaturized and wearable formats [34, 35]. Optical and fluorescence-based tests, while highly sensitive, typically require labeling procedures, fluorophores, or enzymatic reagents that increase procedural complexity and cost [36, 37]. Molecular diagnostic methods such as PCR and ELISA provide great specificity but rely on wet-lab infrastructure, thermal cycling, and trained technicians, limiting their implementation in decentralized or resource-limited settings [38]. Likewise, imaging technologies such as computed tomography (CT), magnetic resonance imaging (MRI), and positron emission tomography (PET) deliver high anatomical or functional resolution but remain expensive, immobile, and reliant on either ionizing radiation or large-scale instrumentation. By exploiting intrinsic dielectric contrasts in tissues and biomolecules, microwave devices overcome many of these practical limitations and enable direct biochemical and structural interrogation without contrast agents, hazardous radiation, or complex sample preparation. Table 1 summarizes these aspects, outlining the advantages, limitations, and typical applications of microwave technologies relative to established diagnostic modalities.

**Table 1:** Comparative overview of microwave technologies versus conventional diagnostic modalities

Technology	Sample Preparation	Key Advantages	Key Limitations	Typical Applications	References
Biochemical/molecular assays (PCR, ELISA)	Requires reagents, labeling, multi-step workflows.	High sensitivity & specificity; established clinical protocols	Requires reagents & lab; long processing; limited portability	Pathogen detection, biomarker quantification	[38, 51, 52]
Optical/fluorescence methods	Often requires labels or staining.	High spatial resolution; single cell/molecular imaging	Requires labeling; bulky equipment; not ideal for real-time or POC	Microscopy, cell/tissue analysis	[36, 37, 53]
Radiological imaging (CT, MRI)	No biochemical prepapration; sometimes requires contrast agents.	Deep penetration; full-body or organ imaging; high anatomical detail	High cost; radiation (CT); non-portable; not suitable for frequent monitoring	Diagnostic imaging, cancer staging, neuroimaging	[54–56]
Electrochemical sensors	Requires electrode functionalization.	Portable, low-cost, rapid detection in fluids.	Fouling in complex biofluids; limited to liquid samples	Point-of-care fluid diagnostics (e.g. glucose)	[57–60]
Microwave biosensing (this review)	Label-free; minimal preparation.	Non-ionizing; label-free; potential for portable/wearable devices; real-time dielectric sensing	Indirect measurement; sensitive to physiological/environmental variables; many applications still proof-of-concept	Tissue characterization, fluid monitoring, point-of-care sensing	[11, 14, 61, 62]

Beyond diagnostics, microwave technology is rapidly emerging as a versatile platform for various therapeutic applications, with recent advances demonstrating that nanotechnology-assisted microwave therapy can significantly enhance heating efficiency, tumor selectivity, and theranostic performance [39]. These therapeutic approaches leverage the unique ability of electromagnetic fields to penetrate biological tissues and modulate physiological processes with high spatial and temporal resolution. These interactions can be used to produce specific temperature effects, stimulate biochemical responses, or modify the behavior of excitable tissues, all while limiting invasiveness and systemic impacts [40]. Microstrip-based emitters can provide controlled temperature gradients in the 2–6 GHz range, enabling applications such as thermal ablation, drug delivery, and tissue regeneration [41–45]. Thermo-responsive polymeric systems integrated into these platforms can release therapeutic compounds when needed, allowing for smart drug delivery methods that are coupled with real-time physiological data. Furthermore, the development of wearable and implantable microwave antennas has opened new avenues for neuromodulation, in which pulsed electromagnetic stimulation can selectively activate or inhibit neural circuits, providing non-pharmacological treatments for chronic pain, depression, epilepsy, and neurodegenerative disorders [45–50]. The flexibility to customize frequency, power, and waveform parameters also enables personalized therapy procedures, which enhance efficacy and safety in various clinical settings.

The novelty of this review lies in providing an integrated and multiscale perspective that unifies dielectric fundamentals, molecular biosensing, cellular and tissue-level microwave characterization, and therapeutic applications within a single coherent framework. Unlike conventional reviews focused on isolated subfields, this work emphasizes the transversal role of advanced functionalities, including microfluidics, wearable and implantable platforms, AI-assisted data processing, and Internet of Medical Things (IoMT)-enabled architectures, as key enablers bridging diagnostic and therapeutic microwave technologies. By consolidating these dimensions with a particular focus on microstrip-based systems, this review delivers a comprehensive synthesis that highlights emerging synergies, technological convergence, and the translational potential of next-generation microwave biomedical platforms.

Building on this framework, the review provides a comprehensive and critical assessment of recent advances in microwave technologies for biomedical diagnosis and therapy, with a particular emphasis on microstrip sensor platforms as versatile and scalable implementations. After the present introduction, Sect. 2 outlines the fundamental principles of dielectric characterization in biomaterials, establishing the biophysical basis for microwave biosensing, imaging, and therapeutic energy delivery. Section 3 surveys microwave approaches for biomarker detection, organized by pathological domain. Section 4 addresses microstrip sensors for cellular and tissue characterization, with emphasis on oncology and inflammatory disease, where microwave diagnostics are demonstrating concrete clinical relevance. Section 5 examines microwave-based therapeutic modalities such as hyperthermia and ablation, covering both physical foundations and recent clinical developments. Section 6 discusses advanced functionalities including microfluidics, portability, machine-learning algorithms, and multiparametric IoMT-connected platforms—that act as cross-cutting enablers across all device classes. Finally, Sect. 7 synthesizes the main challenges and knowledge gaps and outlines future perspectives toward fully integrated microwave theranostic platforms within precision medicine.

## Fundamentals of dielectric characterization in biomaterials

This section provides only the dielectric concepts required to interpret platform-level trade-offs (sensitivity, depth, SAR/temperature prediction, and reproducibility) discussed in Sects. 3–7.

### Basic concepts: permittivity and loss tangent

Dielectric characterization underpins the performance of microwave-based biomedical platforms because wave propagation, energy deposition, and thermal response are directly governed by tissue permittivity and losses. Unlike idealized media, biological tissues behave as dispersive and heterogeneous dielectrics, which fundamentally constrain sensitivity, depth, and reproducibility.

This behavior is commonly described through the complex relative permittivity, defined as [63]:

1 $$\varepsilon^\ast=\varepsilon^{'}\:-j\varepsilon^{''}$$

where $\:\epsilon\:{\prime\:}$ and $\:\epsilon\:{\prime\:}{\prime\:}$ directly determine resonance shifts, attenuation, and electromagnetic energy dissipation in sensing, imaging, and therapeutic systems. A frequently used derived parameter is the loss tangent (calculated using Eq. (2)), which provides a compact metric linking dielectric losses to heating efficiency, field attenuation, and SAR distribution in therapeutic microwave applications [63].

2 $$\:tan\delta\:=\frac{\epsilon\:''\:}{{\epsilon\:}^{'\:}}$$

The loss tangent is therefore a key parameter for estimating heating efficiency and uncertainty in specific absorption rate (SAR) predictions.

The dielectric response of biological tissues arises from multiple polarization mechanisms acting across different spatial and temporal scales, including ionic conduction, Maxwell–Wagner interfacial polarization, and water dipolar relaxation. These processes are commonly summarized by the α-, β-, and γ-dispersion regions, which provide a practical framework for interpreting dielectric spectra in biological media beyond homogeneous-material assumption [64].

Hydration, electrolyte concentration, membrane integrity, and temperature modulate these dispersion mechanisms, producing measurable dielectric contrast across tissues and disease states [65–67]. However, this sensitivity also introduces variability, as ε′ and ε″ are dynamic quantities that fluctuate across subjects and over time, often dominating baseline drift and limiting inter-platform reproducibility.

To account for these multiscale relaxation phenomena, biological permittivity is frequently modeled using generalized formulations such as the Cole–Cole model:

3 $$\:{\epsilon\:}^\ast\left(\omega\:\right)={\epsilon\:}_{\infty\:}+\sum\:_{k=1}^N\frac{\triangle{\epsilon\:}_k}{1+\left(j\omega\:{\tau\:}_k\right)^{1-{\alpha\:}_k}}+\frac{\sigma\:}{j\omega\:{\epsilon\:}_0},$$

where $\:{\epsilon\:}_{\infty\:}$ is the permittivity at infinite frequency, $\:{\Delta\:}{\epsilon\:}_{k}$ is the relaxation strength, $\:{\tau\:}_{k}$ the characteristic relaxation time, $\:{\alpha\:}_{k}$ the dispersion broadening parameter, and $\:\sigma\:$ the effective ionic conductivity. Although this formulation captures frequency dispersion and averaged tissue heterogeneity, it relies on effective parameters and cannot fully represent spatial anisotropy, perfusion-driven thermal transport, or microscale structural variability present in real biological environments.

These modeling limitations directly impact biomedical microwave applications. In sensing and imaging, oversimplified dielectric assumptions propagate into baseline drift, reduced specificity, and limited reproducibility, whereas in hyperthermia and ablation they lead to inaccurate predictions of SAR and temperature distributions, with direct consequences for safety margins and therapeutic efficacy [68–74]. Consequently, contemporary biomedical microwave design increasingly emphasizes frequency selection, rigorous calibration strategies, validated dielectric phantoms, and adaptive modeling approaches that explicitly account for physiological variability and measurement uncertainty. These frequency-dependent dielectric behaviors provide the physical basis for microwave-based diagnostic contrast and motivate the need for accurate dielectric characterization in biomedical diagnosis, as discussed in the following section.

###  Importance of dielectric characterization for biomedical diagnosis

Optical modalities provide high spatial resolution but become increasingly depth-limited in biological tissues due to scattering and absorption. In contrast, microwave and millimeter-wave techniques probe dielectric relaxation and ionic conduction mechanisms linked to water content and tissue conductivity, enabling volumetric contrast at depths where optical performance degrades [75–79]. This complementarity motivates dielectric characterization as a label-free route for assessing tissue state and guiding frequency selection in microwave-based diagnosis, which constitutes the primary focus of this review.

In biosensor development, dielectric characterization provides a practical pathway to translate physiological and pathological alterations into measurable electromagnetic observables. Variations in permittivity and conductivity manifest as resonance shifts, attenuation changes, or phase perturbations, forming the basis for microwave biosensing across the 300 MHz to 300 GHz range, where frequency-dependent dispersion governs diagnostic contrast.

Crucially, dielectric characterization is not merely descriptive but directly informs system design and diagnostic reliability. Identifying frequency windows where dielectric contrast is maximized, it is essential for achieving reproducible and interpretable measurements. In this regard, frequency selection represents a trade-off between penetration depth, sensitivity to specific biophysical mechanisms, and robustness against physiological variability.

Understanding these spectral dependencies is essential not only for diagnostic sensitivity but also for interpretability and robustness. Rather than serving as a general electromagnetic taxonomy, Table 2 summarizes dominant dielectric mechanisms from a design-oriented perspective, illustrating how frequency selection influences penetration depth, contrast generation, and diagnostic reliability.

**Table 2:** Electromagnetic frequency bands, dominant dielectric mechanisms, and representative biomedical applications. The classification highlights how specific interactions with physiological phenomena inform the selection of optimal frequency ranges for diagnostic and therapeutic purposes

Frequency Band	Dominant Mechanism	Typical Application	Reference
< 1 kHz	Interfacial polarization	Membrane studies and neural tissue analysis	[80, 81]
1 kHz – 10 MHz	Dipolar polarization (β‑dispersion)	Electrophysiology and muscle stimulation	[82, 83]
10 MHz – 3 GHz	Dipole rotation (water)	Tissue hydration sensing, glucose monitoring	[84, 85]
3–30 GHz	Water relaxation, bound-water polarization	Dielectric spectroscopy, cancer biomarker detection	[24, 86, 87]
30–100 GHz	Rotational modes, high-frequency relaxation.	Skin hydration, non-invasive thermal imaging.	[86, 88]
100–300 GHz	Collective vibrational modes, lattice oscillations	High-resolution imaging, molecular fingerprinting	[89–91]

Because microwave contrast arises directly from frequency-dependent variations in ε′ and ε″, even modest inaccuracies in their estimation can propagate into significant diagnostic errors, affecting resonance shifts, tissue-contrast interpretation, and quantitative feature extraction. Consequently, dielectric characterization is not only fundamental for understanding biophysical interaction mechanisms but also for ensuring that microwave biosensing and imaging systems operate within frequency regimes that maximize pathological contrast while maintaining quantitative reliability and clinical robustness.

### Microwave methods for material characterization

Microwave dielectric characterization methods can be broadly grouped into non-resonant, resonant, and metamaterial-based approaches according to their electromagnetic interrogation strategy and tolerance to biological heterogeneity [92]. This classification highlights how different measurement principles condition bandwidth, spatial selectivity, and susceptibility to physiological variability in practical characterization scenarios. A comparative summary of these microwave characterization techniques, including their operating principles, advantages, and limitations, is presented in Table 3.

**Table 3:** Comparative summary of microwave characterization techniques

Method	Operating Principle	Typical Samples	Main Advantages	Limitations
Free-space (non-resonant)	Plane-wave transmission/reflection between antennas	Bulk solids, large tissues	Non-contact, very broadband	Sensitive to alignment, diffraction
Open-ended coaxial probe (non-resonant)	Fringing fields at the probe tip interacting with material	Liquids, tissues, semi-solids	Fast, in-situ, small sample	Sensitive to air gaps, limited for inhomogeneous materials
Transmission line method (non-resonant)	Changes in propagation constant in filled waveguides	Solid bars, pellets	Accurate bulk properties	Requires precise sample geometry
Microstrip/CPW/SIW resonators (line-based resonant)	Resonance shift + Q-factor change	Thin films, droplets, phantoms	High sensitivity, small samples	Narrowband, fabrication-dependent
Metamaterial resonators (SRR/CSRR/DSRR/ICR)	Subwavelength field confinement & engineered dispersion	Surface layers, microfluids	Extreme localization, high sensitivity	High design complexity

Non resonant techniques provide broadband access to dielectric dispersion and are therefore suited for characterizing highly hydrated or structurally irregular biological samples when averaged tissue properties are of interest. Resonant methods exploit localized field confinement so that small dielectric perturbations produce measurable shifts in resonance frequency and quality factor, enabling enhanced sensitivity to subtle compositional or structural changes. However, this sensitivity is inherently associated with reduced bandwidth and increased susceptibility to environmental fluctuations and fabrication tolerances.

Metamaterial and planar resonator architectures further enhance field localization through engineered subwavelength confinement, increasing energy density in regions of interest and enabling the detection of weak dielectric signatures that may not be resolved by conventional resonators [12]. The resulting gain in sensitivity is accompanied by higher design complexity and stronger dependence on precise geometrical and material control.

In biomedical applications, the selection of a characterization technique must therefore be guided by the spatial scale of interest, the expected dielectric contrast, and the dominant relaxation mechanisms associated with the targeted physiological or pathological process. No single method is universally optimal. Instead, appropriate technique selection balances sensitivity, penetration depth, bandwidth, and measurement robustness in relation to the specific diagnostic or translational objective.

#### Non-resonant techniques

Non resonant techniques enable broadband dielectric characterization by directly capturing frequency dependent dispersion behavior in biological tissues. This capability makes them particularly suitable for identifying relaxation processes across wide spectral ranges and for characterizing samples whose size, hydration level, or geometry preclude the use of confined resonant structures [93].

From a biomedical perspective, these methods are best suited for bulk or averaged tissue characterization, including hydrated tissues, ex vivo specimens, and in situ measurements where strict sample preparation is impractical. Their principal limitation lies in the lack of spatial selectivity, as the measured response represents an average over relatively large interaction volume [94, 95].

##### Free space method

**Fig. 1:**
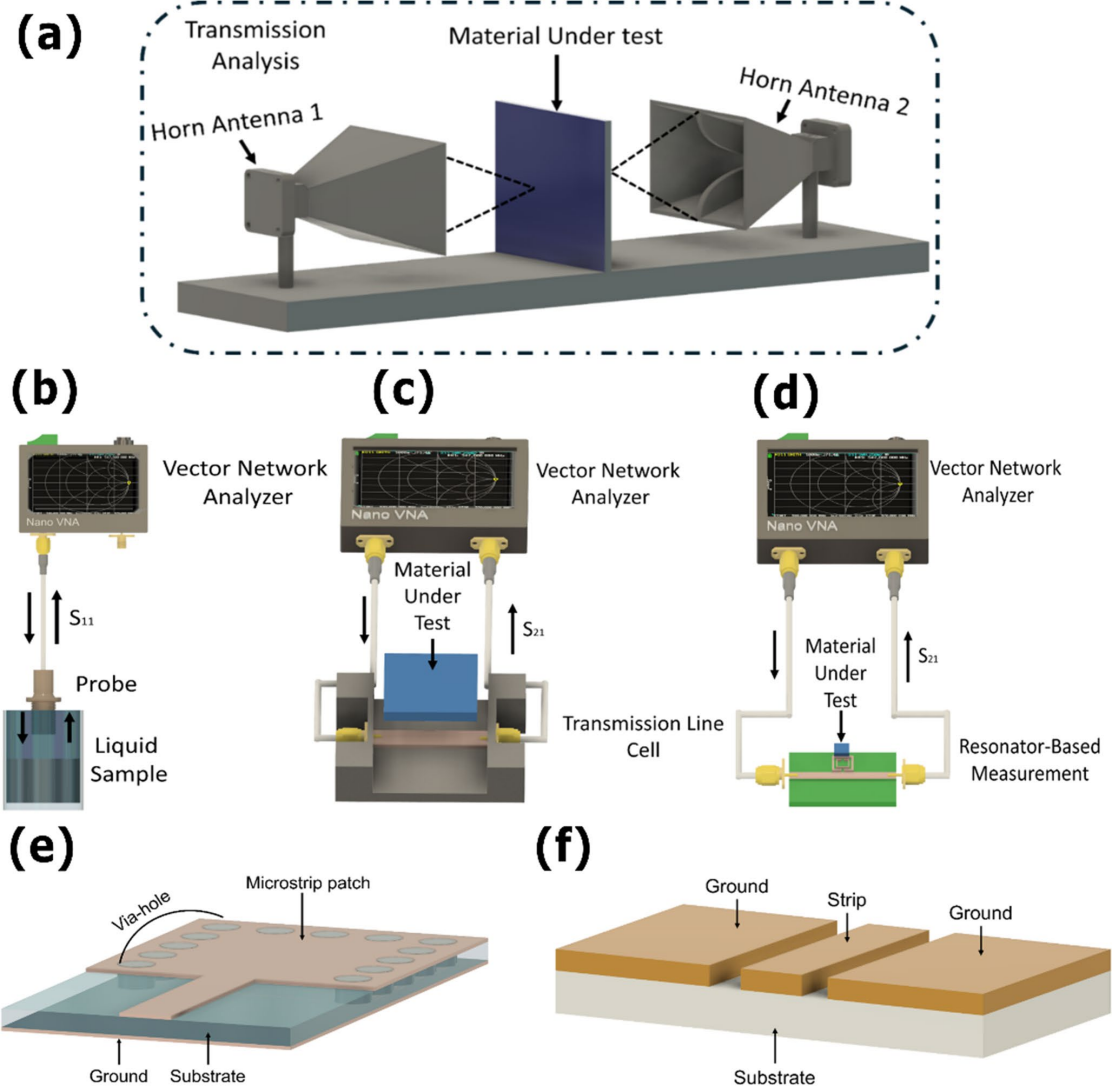
Schematic representations of different microwave characterization setups: (a) Free-space measurement setup with two horn antennas aligned and the sample positioned between them. (b) Open-ended coaxial probe setup. (c) Transmission line method setup. (d) Resonance-based method using an split ring resonator (SSR) microwave resonator for dielectric characterization. (e) A coplanar waveguide (CPW) resonator, consisting of a central conducting strip flanked by two ground planes separated by lateral slots on the same substrate. (f) Substrate integrated waveguide (SIW) resonator.

The free-space method is a non-contact and non-destructive technique widely used for microwave dielectric characterization when samples are large, irregularly shaped, or incompatible with guided-wave fixtures [96–98]​. Figure 1a shows a material under test is illuminated by a pair of horn antennas, and changes in transmitted and reflected signals are used to extract effective dielectric properties over a broad frequency range.

Its main strength lies in broadband operation combined with the absence of physical contact, which enables characterization of bulk tissues and heterogeneous specimens with minimal sample preparation. However, measurement accuracy is strongly influenced by alignment errors, surface roughness, diffraction, and multipath scattering. In biological tissues, where hydration gradients and irregular boundaries are intrinsic, these effects introduce uncertainty that can obscure subtle dielectric contrasts.

As a result, free space measurements are well suited for estimating bulk dielectric behavior and for validating dielectric models, but they lack the spatial sensitivity required to resolve microscale anomalies. In biomedical research, they are therefore typically used for baseline characterization rather than for high sensitivity diagnostic sensing, which generally requires localized field confinement [99, 100].

##### **Open-ended coaxial probe** 

The open-ended coaxial probe is a widely used non-destructive technique for broadband dielectric characterization of liquids, semi solids, and hydrated biological tissues [101, 102]​. It operates through fringing electric fields generated at the open tip of a coaxial line placed in contact with the sample, enabling broadband extraction of effective complex permittivity with minimal sample preparation​.

A schematic of the experimental setup is shown in Fig. 1b. Its principal advantages are measurement speed, portability, and compatibility with in situ or ex vivo tissue assessment, making coaxial probes suitable for rapid screening and preliminary dielectric evaluation of hydrated biological media. However, the effective interaction volume is confined to the immediate vicinity of the probe tip, causing the measured response to be dominated by surface hydration and boundary conditions rather than bulk tissue properties.

In biological samples, tissue deformation under contact, air gap formation, and local heterogeneities introduce systematic errors that limit sensitivity to weak or localized dielectric variations. Consequently, open ended coaxial probes are primarily employed for reference measurements or validation purposes, while the detection of subtle pathological or biochemical changes generally relies on resonant or metamaterial-based platforms capable of enhancing dielectric contrast [95].

##### Transmission line method

The transmission line method determines dielectric properties by inserting the material under test into a guided structure and analyzing changes in phase and attenuation relative to a reference condition ​ [103–106]. Because the electromagnetic field propagates through the entire sample cross section, this approach provides accurate estimates of bulk permittivity over broad frequency ranges, particularly for materials with moderate to high dielectric losses.

Its applicability to biological tissues is, however, strongly constrained by geometric and boundary condition requirements. Biological samples cannot be reproducibly shaped or compacted without altering hydration, microstructure, or physiological state, and their intrinsic heterogeneity violates the assumptions of uniform field interaction underlying transmission line analysis. As a result, this method is rarely used as a direct sensing platform in biomedical contexts.

In practice, transmission line techniques serve primarily as calibration references or for validating dielectric models using engineered or homogeneous materials. Their strength lies in controlled material characterization rather than in accommodating the variability and structural complexity inherent to living tissues.

#### Transmission line resonant techniques

Transmission line resonant techniques exploit localized field confinement to enhance sensitivity to small dielectric perturbations, enabling detection of subtle compositional or structural changes that are not accessible through broadband averaging methods.

##### Microstrip resonators

Microstrip resonators characterize dielectric properties by monitoring shifts in resonant frequency and quality factor induced by the presence of a material within the sensing region [21, 107–109]. Their operation relies on localized electromagnetic field confinement, so that small variations in permittivity and loss directly perturb the resonant condition.

This sensitivity arises from the strong dependence of the resonant condition on the local dielectric environment, where variations in ε′ and ε″ perturb both the effective electrical length and stored electromagnetic energy [80]. From a biomedical perspective, microstrip resonators are well suited for localized sensing scenarios in which spatial selectivity and sensitivity to weak dielectric changes are prioritized over broadband dispersion mapping. Their narrowband nature and susceptibility to environmental drift, however, require careful design, stabilization, and referencing strategies to ensure reliable biosensing performance.

##### Coplanar waveguide (CPW) resonators

Coplanar waveguide resonators confine signal and ground conductors on a single planar surface, producing strong electric field localization near the substrate interface [18, 110–113]. This surface dominated field distribution enables direct interaction with liquids, cells, or functionalized biomolecular layers, making CPW structures particularly suitable for microfluidic integration and surface based biosensing.

Because the quasi-TEM fields are concentrated in the slot region, small dielectric perturbations near the surface produce measurable shifts in resonant frequency. This open planar configuration facilitates direct access to the sensing region without complex sample preparation or substrate modification.

At the same time, CPW resonators are inherently sensitive to environmental fluctuations, including humidity, temperature drift, and unintended surface contamination. Variations in boundary conditions directly affect modal dispersion, leading to baseline instability that can mask weak dielectric signatures. Consequently, CPW based biosensors typically require environmental control, surface passivation, or differential referencing to achieve reproducible biomedical measurements.

##### Substrate integrated waveguide (SIW)

Substrate integrated waveguide resonators are planar structures that replicate the electromagnetic behavior of conventional rectangular waveguides through metallized vias and parallel metal planes, while remaining compatible with standard planar fabrication technologies (Fig. 1f) [114–116]. This quasi-enclosed geometry confines electromagnetic fields within the substrate, resulting in low radiation loss, high quality factor, and strong immunity to external interference.

Because SIW resonators support waveguide like modes with well confined field distributions, dielectric loading of the enclosed region produces reproducible shifts in resonant frequency and quality factor [116, 117]. Compared with open boundary resonators, SIW platforms provide superior baseline stability and reduced sensitivity to environmental fluctuations, including humidity variations, temperature drift, and surface contamination. These characteristics make SIW resonators particularly suitable for characterizing lossy or highly hydrated biological tissues, where measurement robustness and repeatability are critical.

The enhanced field confinement that improves stability also limits interaction with ultrathin surface bound analytes. As a result, SIW resonators typically exhibit lower intrinsic sensitivity to biomolecular layers compared with microstrip or coplanar waveguide configurations. To address this limitation, SIW based biosensors often incorporate perturbation enhancing features such as slots, apertures, or engineered discontinuities to locally increase field exposure and improve sensitivity to surface level dielectric changes.

#### Metamaterial and planar resonator architectures

Metamaterial inspired planar resonators extend conventional resonance-based sensing by enabling engineered dispersion and subwavelength electromagnetic field localization, thereby amplifying the interaction between the resonator and nearby biological media [19, 118, 119]. Rather than relying on global cavity perturbations, these architectures concentrate electromagnetic energy into spatially confined regions, which is critical for resolving weak and highly localized dielectric variations.

**Fig. 2:**
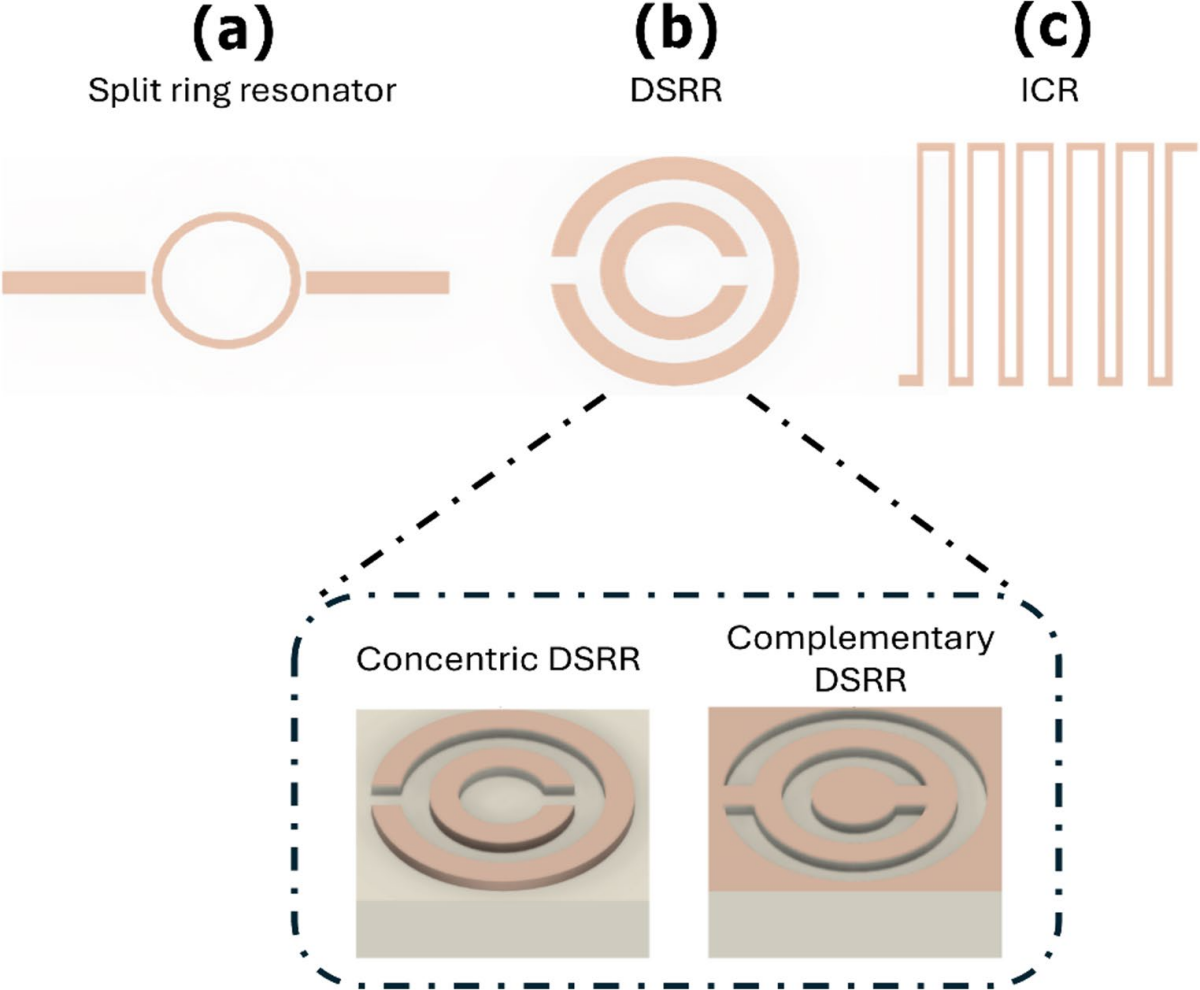
Layout of various electromagnetic resonator geometries: (**a**) Split ring resonator, (**b**) Double split ring resonator (DSRR), and (**c**) Interdigital capacitor resonator (ICR). The zoomed view highlights two DSRR variants: concentric DSRR and complementary DSRR, both etched on a planar dielectric substrate. For a more detailed explanation, please see the main text

In planar microwave biosensing, metamaterial-inspired resonators achieve enhanced sensitivity through patterned metallic geometries that tailor the spatial distribution and symmetry of resonant electromagnetic fields (Fig. 2). Ring resonators (Fig. 2a) primarily respond to global variations in the effective permittivity of the surrounding medium, as their closed-loop geometry produces a distributed field that is sensitive to averaged dielectric changes rather than highly localized perturbations. Split-ring–based geometries introduce localized field discontinuities that selectively enhance sensitivity to specific dielectric components (Fig. 2b). SRR and double split-ring resonators (DSRR) emphasize magnetic-field coupling and are responsive to structural or morphological tissue changes, while the presence of multiple gaps in DSRR configurations further sharpens field localization and resonance selectivity. In contrast, complementary split-ring resonators (CSRR), obtained by etching the resonant pattern into the ground plane, couple predominantly to the electric field and are especially suited for detecting permittivity variations in materials located near or beneath the substrate. Interdigital capacitor resonators (ICR, Fig. 2c) exploit strong fringing electric fields between closely spaced fingers, providing pronounced surface confinement and high sensitivity to superficial dielectric changes associated with hydration shifts, biomolecular binding, or compositional variations within thin biolayers. From a biomedical design perspective, these geometries are not interchangeable: they determine the dominant coupling mechanism (electric versus magnetic), the spatial scale of field localization, and the effective sensing depth, enabling metamaterial platforms to be tailored to specific diagnostic objectives.

At selected frequencies, periodic arrangements of these resonators exhibit collective dispersive responses that sharpen resonance features and improve selectivity, which are exploited to enhance sensing performance rather than to realize bulk metamaterial functionality [113, 120–123]. In biosensing applications, such collective effects are exploited to enhance resonance sharpness and selectivity rather than to realize bulk metamaterial functionality. However, the same field confinement that enables high sensitivity also increases susceptibility to fabrication tolerances, substrate losses, and environmental perturbations, which can degrade baseline stability and reproducibility.

Current research therefore focuses on balancing sensitivity and robustness, through strategies that improve quality factor, reduce environmental drift, and implement multiplexed or differential resonator arrays. These developments are essential for translating metamaterial-based biosensors from laboratory demonstrations into reliable and portable diagnostic platforms suitable for clinical and point of care deployment.

## Molecular-scale microwave biosensing

The detection of biomolecules using microstrip technology presents significant challenges but also offers unique advantages, positioning it as an up-and-coming platform for early disease diagnosis. Among its most attractive characteristics are its inherent label-free functionality, capacity for real-time analysis, seamless compatibility with Internet of Things (IoT) frameworks, and suitability for incorporation into portable point-of-care testing (POCT) devices. These features align with the latest advancements in personalized medicine and decentralized healthcare, where rapid, accurate, and less invasive diagnostic technologies are becoming increasingly necessary.

Despite these advantages, certain limitations must be addressed before microstrip sensors can fully realize their potential. A key difficulty is biological specificity because the electromagnetic response itself cannot distinguish between molecules with comparable dielectric signatures. To address this issue, sensor surfaces are typically functionalized with specific biorecognition layers such as antibodies [124, 125], aptamers [126], or engineered receptors [127, 128]. The performance of these functional coatings is highly dependent on criteria such as molecule orientation, anchoring density, and long-term chemical stability. Inadequate functionalization might result in decreased sensitivity or false positives, reducing diagnostic reliability.

Another significant barrier is the absence of standardized fabrication, calibration, and testing processes. This constraint reduces reproducibility and impedes direct performance comparisons across various sensor designs documented in the literature [16, 129–132]. Furthermore, genuine biological matrices, such as serum, plasma, and saliva, contribute to new sources of variation, including dielectric background interferences, nonspecific molecule interactions, and humidity-induced fluctuations. To address these interfering variables, rigorous calibration procedures, optimal sensor designs, and the use of reference channels or differential measurement schemes are required.

This section focuses on microwave biosensing at the molecular scale. On these platforms, the measurable dielectric perturbation arises from specific biochemical recognition events, such as antigen–antibody binding, aptamer–target interactions, or receptor–ligand complexes occurring at the sensor’s functionalized surface. These localized events modulate the near-field electromagnetic response, enabling selective and label-free detection of disease-related biomolecules. The subsections that follow review the most relevant advances in microstrip molecular biosensors, particularly those targeting cancer biomarkers, where recent designs have demonstrated outstanding sensitivity and specificity.

### Detection of cancer biomarkers

Early cancer detection through molecular biomarkers represents a promising approach to improving clinical outcomes by enabling timelier, less invasive, and more cost‑effective interventions. Microwave‑based biosensor technology offers advantages such as real‑time detection, high dielectric sensitivity to analyte concentration, and label‑free operation, which simplifies the analytical workflow [24, 133]. Various resonant architectures—including SRRs, CSRRs, transmission‑line sensors, and SIW cavities—have been combined with surface-functionalization strategies and specific interface chemistries. These methods have proven significant potential for detecting oncoproteins, tumor antigens, and cancer-associated autoantibodies in colorectal, lung, and breast cancer, among others [113, 133–135].

**Fig. 3:**
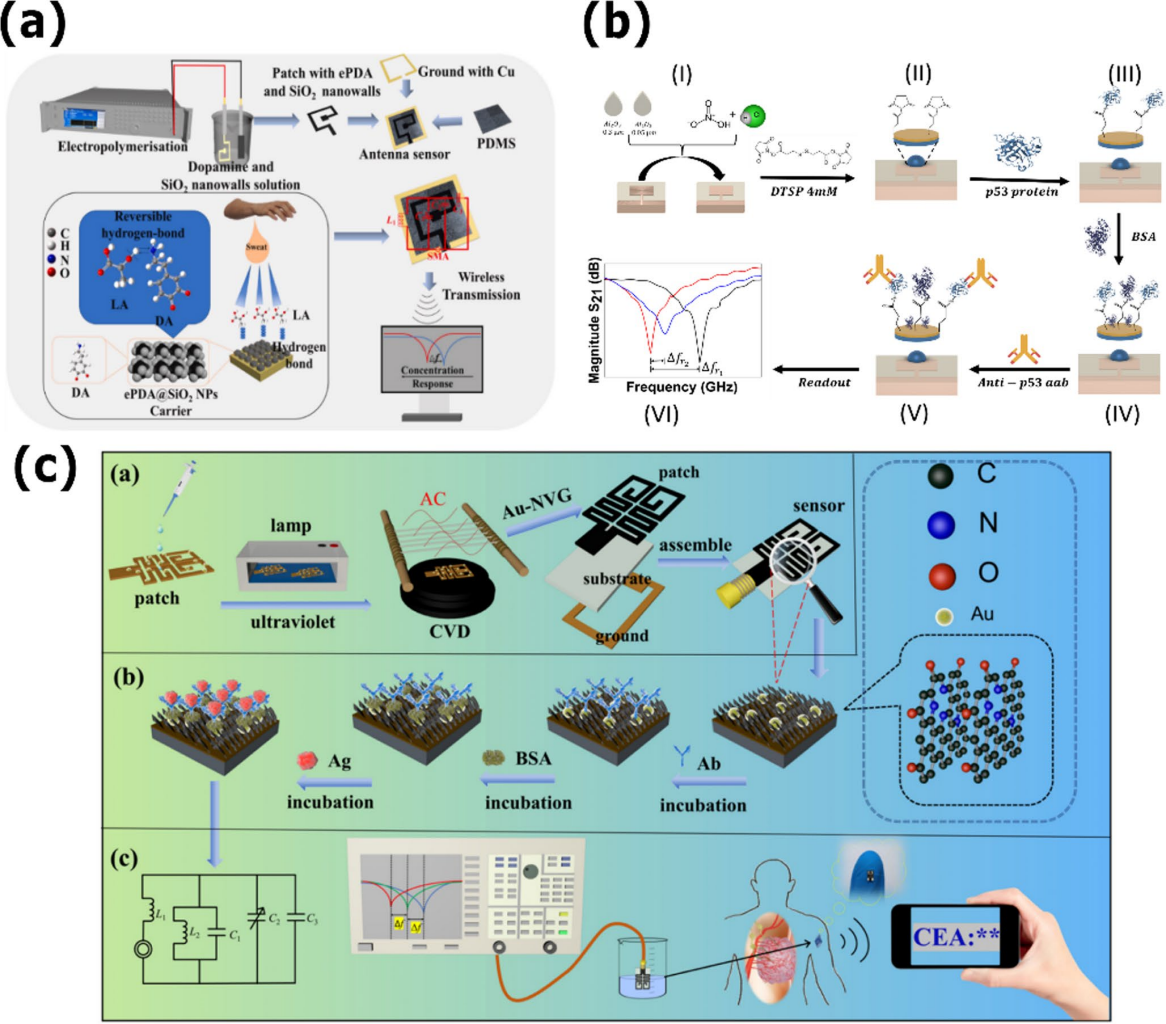
Representative microstrip biosensor architectures and functionalization strategies for biomarker detection. This figure illustrates three distinct approaches for molecular detection via specific surface functionalization: (**a**) a flexible patch antenna coated with ePDA/SiO₂ nanowalls for lactate detection through reversible hydrogen bonds; (**b**) a stepped‑impedance resonator (SIR) surface‑functionalized with DTSP and blocked with BSA to immobilize p53 protein and selectively detect anti‑p53 autoantibodies; (**c**) a miniaturized implantable antenna for wireless carcinoembryonic antigen (CEA) detection, whose surface is covered with a graphene–gold (Au‑NVG) film serving as a platform for anti‑CEA antibody immobilization and antigen capture. Adapted with permission from [24, 87, 136]

As illustrated in Fig. 3, recent advances exemplify three distinct strategies for enhancing the performance of microstrip biosensors through tailored functionalization. Figure 3a depicts a flexible patch antenna coated with electropolymerized polydopamine/silica nanowalls (ePDA/SiO₂) [136], designed for lactate monitoring in biofluids. The nanostructured interface utilizes reversible hydrogen bonding to achieve high sensitivity, while its mechanical flexibility supports wearable and point-of-care (POC) integration. Figure 3b depicts a biosensor based on an SIR structure that was functionalized with 3,3′-ddithiodipropionic acid di(N-hydroxysuccinimide ester) (DTSP) self-assembled monolayers and blocked with bovine serum albumin for selective immobilization of p53 protein, as described by Montoya-Villada et al. [25]. This platform achieved an LOD of 315.29 pg/mL with a sensitivity of 0.105 MHz·(pg/mL) ⁻¹, demonstrating high specificity against interferents such as IL-6 and IL-8, and exhibiting a strong correlation with ELISA assays (R² = 0.97). These results highlight the capacity of microwave sensors to rival gold-standard immunoassays in terms of sensitivity and reliability. Figure 3c presents a miniaturized implantable antenna developed by Chen et al. for wireless detection of CEA [87]. The design incorporates a graphene film decorated with gold nanoparticles as the biorecognition layer, integrated onto a 5 × 3 × 0.127 mm³ radiating patch with an in-body peak gain of − 18.2 dB. This immunosensor achieved a sensitivity of 2.46 MHz·(log(ng/mL)) ⁻¹ over the range of 0.01–100 ng·mL⁻¹, with reproducibility, stability, and serum recoveries ranging from 93.5 to 100.2%, underscoring its promise for continuous in vivo cancer monitoring.

Additional studies further demonstrated the breadth of microwave biosensor applications in oncology. In 2025, Lei et al. developed a microstrip sensor based on an SRR functionalized with anti‑CEA antibodies and coupled to interdigital electrodes [134]. This device achieved a sensitivity of 2.46 MHz·(log(ng/mL))⁻¹ over a concentration range of 0.01–100 ng/mL and a LOD of 0.000318 ng/mL. It also demonstrated excellent specificity, stability over time, and reliable recovery rates in human serum (93.5–100.2%). In breast cancer detection, Bevacqua et al. proposed a compact CSRR-based sensor, evaluated via full-wave electromagnetic simulations [113]. They used electromagnetic simulations to compare their response to dielectric changes in healthy and malignant cells. Their design revealed frequency shifts of up to 133 MHz when probing a 10 mm tumor, achieved an impedance match of S₁₁ = −98 dB with a near-ideal voltage standing wave radio (VSWR) of 1.0005, and demonstrated an average sensitivity of 11.56 MHz·mm⁻¹, emphasizing its ability to discriminate between pathological and healthy tissues with remarkable precision. On the other hand, Lee and colleagues developed an experimental asymmetric SRR (aSRR) transducer for label-free detection of stress biomarkers, including cortisol and α-amylase [21]. The device was fabricated using printed metamaterial elements in a microstrip transmission‑line configuration. For cortisol detection, a competitive assay was employed between free cortisol and cortisol conjugated to bovine serum albumin; α-amylase was detected via direct antigen–antibody recognition. Under experimental conditions, the sensor achieved an LOD of approximately 1 ng/mL for both biomarkers and a sensitivity of up to 1.155 MHz·(ng/mL) ⁻¹for cortisol, enabling rapid response times (< 1 min per cycle) and straightforward fabrication compared to conventional electrochemical sensors.

Collectively, these studies underscore the versatility and adaptability of microstrip-based biosensors for oncological applications. They demonstrate not only competitive detection limits and high selectivity but also the capacity to be tailored through nanostructured coatings, functional self-assembled monolayers, and miniaturized or implantable antenna configurations. Nonetheless, challenges persist, particularly concerning stability in complex biological matrices, dielectric interference from nonspecific biomolecules, and the critical requirement for large-scale clinical validation.

### Detection of inflammatory and metabolic disease biomarkers

Metabolic and inflammatory diseases, such as type 2 diabetes mellitus and its associated cardiovascular conditions, represent one of the leading causes of morbidity and mortality worldwide. In response to these needs, microwave biosensors have emerged as promising tools due to their ability to perform non-invasive, rapid, and reagent-free measurements by exploiting changes in the dielectric properties of biological fluids, such as blood, saliva, or sweat [10]. Their compatibility with portable and wearable form factors, combined with straightforward integration into microfluidic systems and smart textiles, provides a robust framework for continuous monitoring of key metabolic and inflammatory biomarkers, including glucose [137–139], cortisol [21], lactic acid [136], urea [140, 141], sodium [65], immunosuppressive drugs [142], and C-reactive protein (CRP) [9]. These approaches facilitate the development of miniaturized, portable, and wearable-compatible sensors, streamlining integration into telemedicine schemes or POC diagnostics for monitoring clinically relevant metabolites [136–141].

An early example is the ultra-sensitive inductor and a capacitor **(**LC) resonator on a GaAs substrate introduced in [143], which exhibited a frequency sensitivity of 117.5 MHz·(mg/mL)⁻¹ to glucose solutions, with near-perfect linearity (R² = 0.9987), amplitude variation of 0.49 dB·(mg/mL)⁻¹, and reproducibility below 1% relative standard deviation. Similarly, a CSRR-based biosensor developed for hemodialysis monitoring achieved non-invasive urea quantification with a detection range of 1–100 mg/dL, sensitivity of 0.021 MHz·(mg/dL) ⁻¹, and a central operating frequency of 2.4 GHz, closely matching the physiological blood urea range (15–51 mg/dL) [141].

Wearable and microfluidic innovations have further expanded the translational potential of microwave biosensors. In 2025, Hussain Mian and collaborators presented a textile-embedded microwave biosensor for continuous, non-invasive glucose monitoring in the sweat of patients with type 2 diabetes [10]. Operating at 4.87 GHz, it delivered a sensitivity of 0.092 (mg/dL) ⁻¹, a quality factor (Q) of 240, and a maximum error of 3.1% compared to a commercial clinical meter (GREEN-DOCTOR G400). In parallel, a microfluidic immuno-biosensor integrating an ELISA chip with a microwave micro-spectrometer enabled highly sensitive quantification of hs-CRP in urine—a key biomarker of inflammation and renal damage—with an LOD of 0.1 ng/mL, calibration ranges spanning 1–1500 ng/mL, and recoveries up to 106.2% in samples from 41 chronic kidney disease patients [9].

**Fig. 4:**
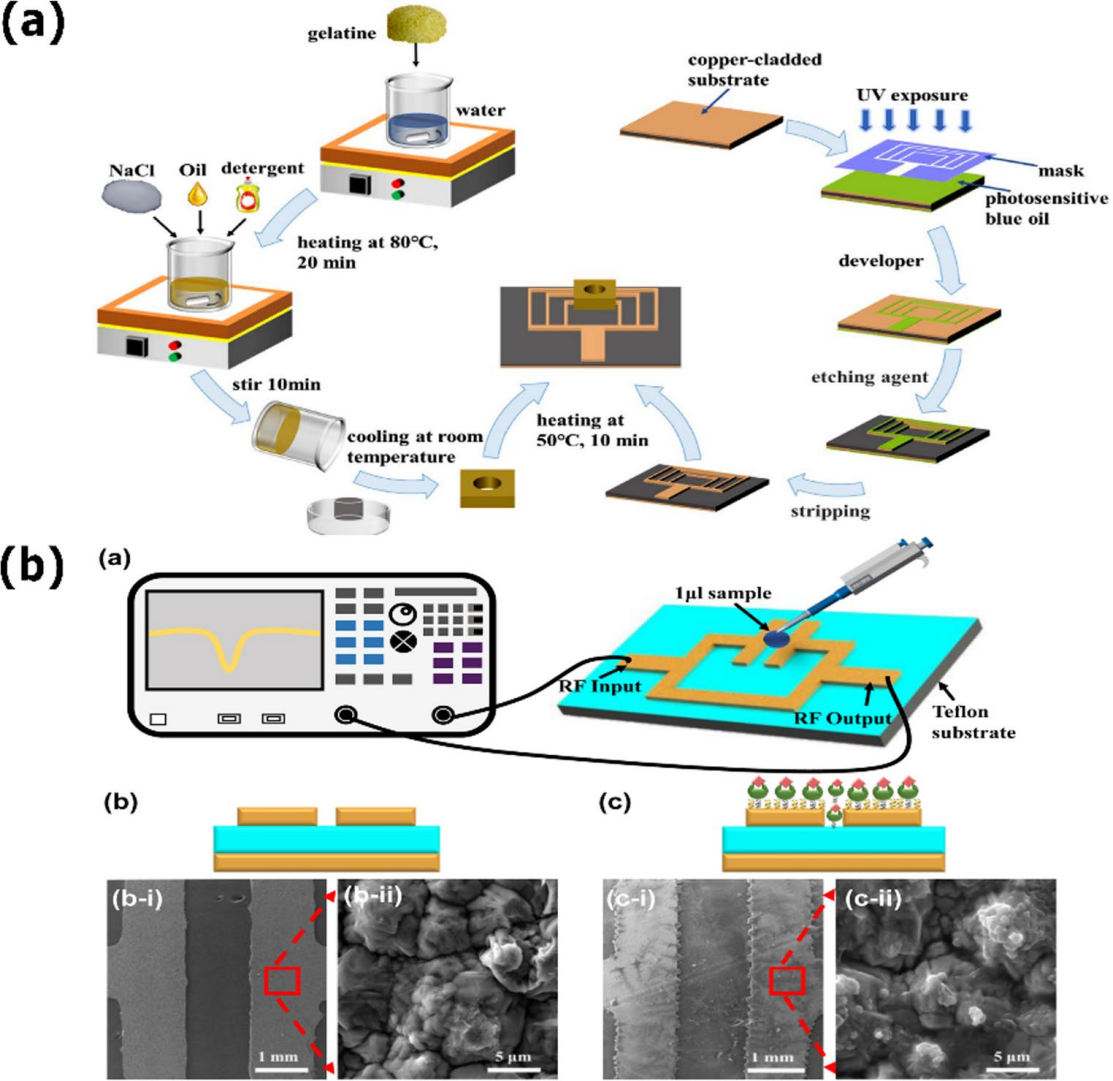
Representative examples of microwave biosensors for metabolite and drug monitoring. The figure illustrates three distinct architectures and applications: (**a**) a laboratory‑scale triplering resonator integrated with a biomimetic microfluidic channel for precise glucose quantification in controlled sample volumes; (**b**) a planar SRR‑based sensor for therapeutic drug monitoring of the immunosuppressant tacrolimus, highlighting its relevance to personalized medicine. Adapted with permission from [142] and adapted from [144] licensed under CC BY 4.0

Further work has optimized resonator architectures for metabolic sensing. Omer et al. proposed a non-invasive microwave biosensor based on a triple-pole CSRR for real-time glucose monitoring in fluids simulating clinical concentrations [123]. The device operates within the 1–6 GHz band and was validated with solutions mimicking glucose levels of 70–120 mg/dL. Transmission measurements yielded a sensitivity of 2 dB·(mg/mL) ⁻¹, demonstrating a high capability to detect dielectric permittivity changes associated with typical variations in human plasma glucose levels. Zhang et al. developed a triple-ring microstrip biosensor with a biomimetic microfluidic channel (Fig. 4a) for precise glucose quantification in simulated solutions [144]. The system comprises three concentric ring resonators patterned on a Teflon substrate, connected to a polydimethysiloxane (PDMS) channel that guides 1 µL sample volumes. During characterization, the sensor exhibited a sensitivity of 0.25 MHz·(mg/dL) ⁻¹, a LOD of 7.7 mg/dL, and an operational range of 50–500 mg/dL. The correlations between resonance frequency and glucose concentration reached R² = 0.996 for the frequency shift and R² = 0.984 for the reflection coefficient. Additionally, a 150 ms response time was reported, reinforcing its suitability for early and rapid glucose monitoring in portable and POC applications. Moving toward therapeutic monitoring, Yang et al. reported a planar microwave SRR-based biosensor (Fig. 4b) for the quantitative detection of tacrolimus in transplant patients [142]. The device achieved a sensitivity of 32.37 MHz·(ng/mL) ⁻¹ and an LOD of 0.032 ng/mL, validated in both aqueous and real blood samples with high linearity (R² = 0.99).

### Detection of neurodegenerative disease biomarkers

Neurodegenerative disorders such as Alzheimer’s disease, Parkinson’s disease, and amyotrophic lateral sclerosis impose an ever‑growing clinical and societal burden, particularly in aging populations. These disorders are characterized by the progressive and irreversible loss of neuronal function, for which no curative treatment currently exists; therefore, the early detection of associated biomarkers is essential. Conventional diagnostic methods, such as magnetic resonance neuroimaging or protein assays in cerebrospinal fluid, are limited by high operational costs, accessibility constraints, and invasiveness [145–147]. Consequently, there is increasing interest in non‑invasive technologies capable of monitoring dielectric property changes in biological tissues, fluids, or intracellular structures [35]. This approach enables the detection of functional alterations related to mitochondrial dysfunction, misfolded protein accumulation, or structural deterioration, hallmarks of early neurodegenerative disease stages [148].

**Fig. 5:**
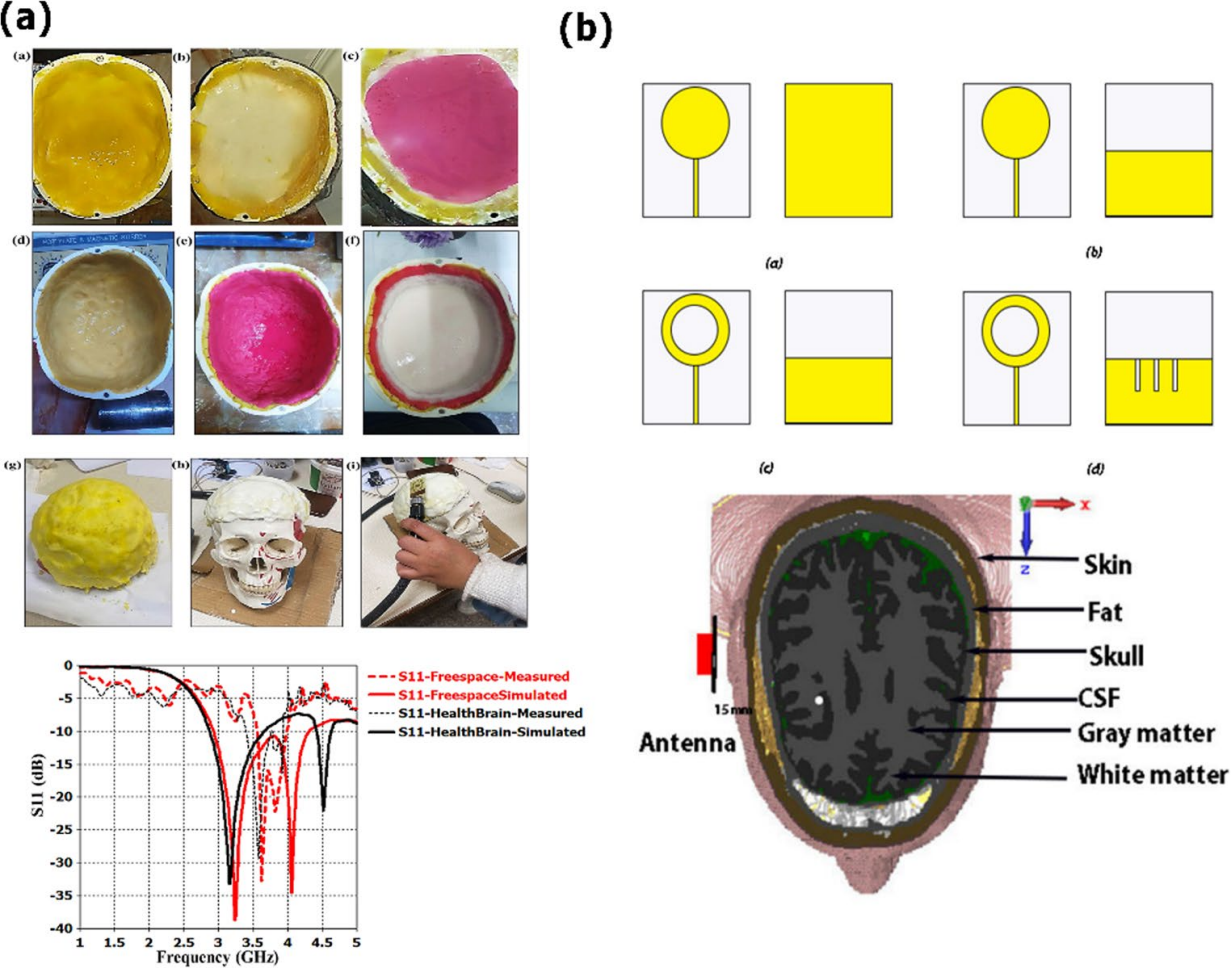
Microwave sensor designs for Alzheimer’s disease detection via dielectric characterization of brain tissue. (**a**) Multilayer head phantom model incorporating the hippocampus as the first region affected by Alzheimer’s disease. The phantom, designed in Computer Simulation Technology Studio (CST) with realistic dielectric properties for skin, fat, skull, cerebrospinal fluid, gray matter, white matter, and hippocampus, was used to evaluate stage-dependent changes. Simulated and experimental reflection coefficient (S₁₁) responses showed resonance shifts near 3 GHz and magnitude variations greater than 12 dB between healthy and Mild Cognitive Impairment due to Alzheimer’s Disease (MCI-AD) models, demonstrating sensitivity to hippocampal atrophy and dielectric alterations. (**b**) Evolutionary design and validation model for an ultra‑thin, wearable UWB antenna. The top row illustrates four design stages—from a simple circular patch to the final structure featuring a circular slot in the patch and three rectangular slots in the partial ground plane—optimized for enhanced bandwidth and efficiency. The bottom row depicts the six-layer brain phantom (skin, fat, skull, cerebrospinal fluid, gray matter, white matter) used to simulate antenna-tissue interaction and validate its capability to monitor cognitive impairment–related changes. Adapted from [148, 150] licensed under CC BY 4.0

At the subcellular scale, microwave dielectric spectroscopy (MWDS) has been utilized as a label-free technique to detect mitochondrial membrane depolarization in living cells [149]. In that study, the dielectric contrast Δ𝜀′ was measured at 5 GHz, and exposure to electron-transport-chain inhibitors (carbonyl cyanide-p-trifluoromethoxyphenylhydrazone (FCCP), and Antimycin A) induced significant increases in Δ𝜀′. These microwave-derived changes closely correlated with flow-cytometry-verified loss of mitochondrial potential, positioning MWDS as a viable tool for real-time, non-invasive functional monitoring of early neuronal pathology. Focusing on non-invasive head sensing, Shehab et al. [150] proposed a compact diamond-shaped ultra-wideband (UWB) antenna integrated into a multilayered realistic head phantom for Alzheimer’s stage differentiation (Fig. 5a). The sensor, with dimensions of 32 × 37 × 0.36 mm³, operates at 3.241 GHz in simulations and 3.62 GHz in experiments, covering an effective band between 2 and 4 GHz. It achieved a bandwidth of 1.4 GHz, a gain of 1.56 dB, and a SAR of 0.566 W/kg, ensuring deep tissue penetration while remaining within safety standards. The authors developed six- and seven-layer head phantoms that accurately mimic the anatomical and dielectric properties of the brain, explicitly including the hippocampus as the first affected region in AD progression. Experimental validation using a vector network analyzer confirmed sensitivity to disease-related dielectric variations, with observable shifts in resonance frequency (up to 80 MHz) and return loss (ΔS₁₁ > 12 dB) across mild, moderate, and severe stages.

Complementarily, an ultra-thin wearable slotted-disk UWB antenna (Fig. 5b) has been proposed for cognitive monitoring [148]. Operating in the UWB band with an adequate bandwidth of 8.55 GHz (2.02–10.57 GHz) and a peak gain of 3.1 dBi, the device maintains SAR levels within safety limits (0.734 W/kg at 2.4 GHz). While not directly targeting molecular biomarkers, this platform enables continuous, real-time monitoring of brain functionality, offering potential for portable detection and follow-up of Alzheimer’s disease. Beyond localized sensors, microwave brain imaging (MBI) systems have also been explored. A notable contribution by Hossain et al. introduced a compact UWB antenna array consisting of 16 elements arranged in a circular pattern around a 3D head phantom [151]. Operating between 1.2 and 3 GHz, the system employed a Delay-and-Sum reconstruction algorithm to detect simulated tumors as small as 2 cm in diameter, with localization errors of less than 3 mm. Moreover, the system successfully distinguished tumors of varying dielectric contrasts, confirming its sensitivity to subtle dielectric variations. This work highlights the potential of microwave-based imaging as a complementary, accessible, safe, and portable diagnostic tool to MRI. Thus, these investigations demonstrate the multi-scale adaptability of microwave technologies in neurodegenerative disease diagnostics, ranging from examining mitochondrial malfunction at the cellular level to tracking disease development in the brain.

### Detection of bacterial and pathogenic biomarkers

Another viable approach for label-free detection of bacterial agents involves exploiting dielectric changes induced by microorganisms or their metabolites using microstrip platforms. Although bacteria and fungi are whole cells, the microwave biosensors reviewed here operate within a pathogen-detection framework that is conceptually aligned with molecular biosensing rather than cellular or tissue dielectric characterization. In these systems, the electromagnetic response is governed by localized surface interactions, such as antigen–antibody binding, adsorption onto functionalized interfaces, or dielectric perturbations induced by metabolic by-products, rather than by the volumetric dielectric properties of intact microbial cells.

Bacterial and fungal infections are a significant cause of morbidity and mortality worldwide, especially in clinical‑hospital settings and among immunocompromised patients. Early detection of pathogenic agents is crucial in preventing the spread of transmissible diseases. Conventional methods, such as microbiological cultures or PCR assays, while effective, require lengthy processing times, specialized personnel, and stringent laboratory conditions. In response to these limitations, microwave sensors have emerged as a promising rapid‑detection alternative by leveraging variations in the dielectric properties of microorganisms or their components to identify pathogenic species via S‑parameter measurements [152–156].

**Fig. 6:**
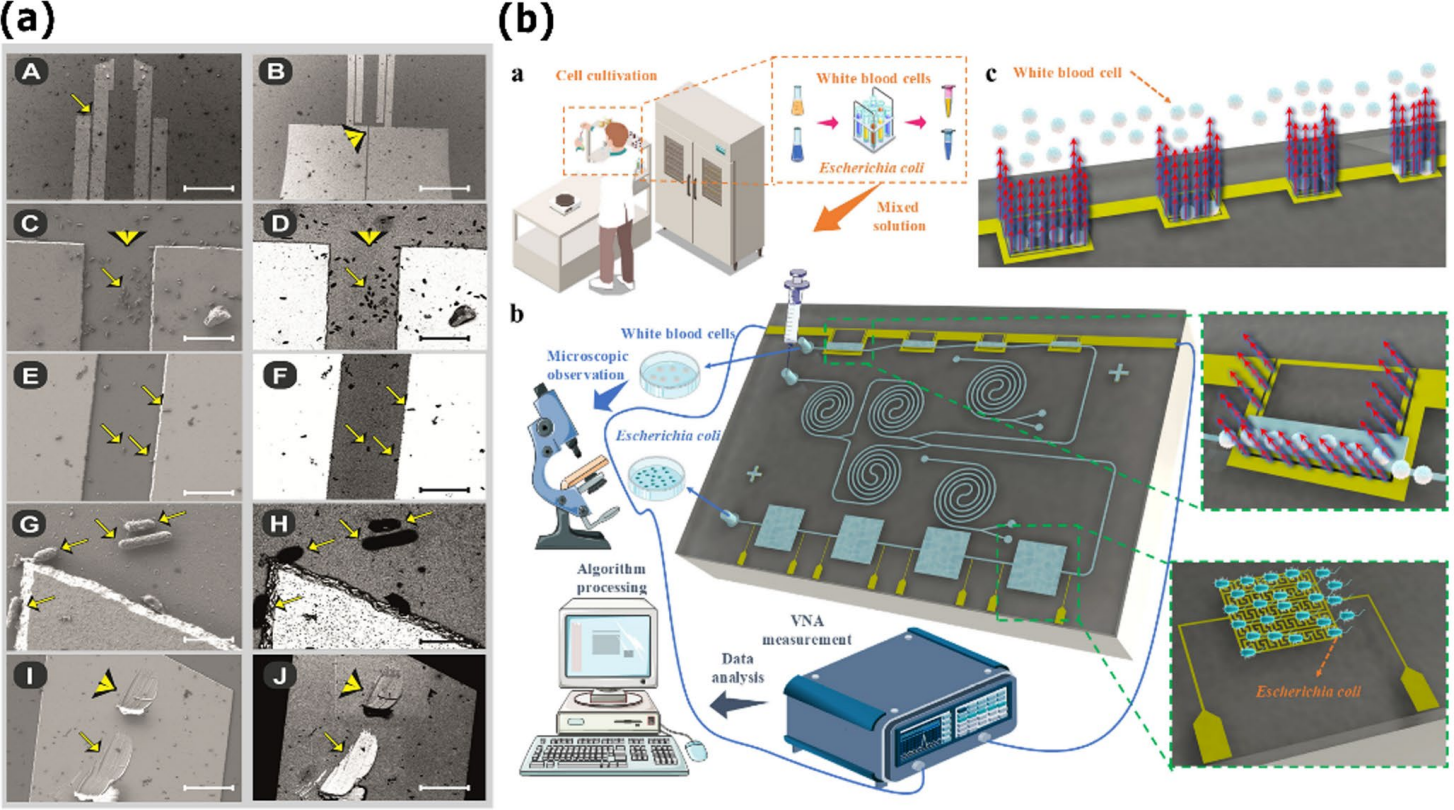
Microwave biosensor approaches, bacterial pathogen detection, and processing. (**a**) Scanning electron micrographs (SEM) confirming successful immobilization of *E. coli* on the surface of an antibody‑functionalized microwave sensor. Images at two magnifications (scale bars: 10 and 2 μm) highlight the bacteria (arrows) attached to the resonator electrodes, demonstrating the effectiveness of the specific capture strategy—a critical step for selective detection. (**b**) Schematic of an integrated microfluidic–microwave system for the separation and detection of *E. coli* in mixed samples containing white blood cells (WBCs). The platform employs a spiral microchannel for inertial enrichment and separation of cells into distinct outlets, followed by interrogation with microstrip resonators. Machine–learning–based classification of the resulting S₁₁ response enables label‑free analysis of complex biological samples. Adapted from [13, 155] licensed under CC BY 4.0

Various microwave-based biosensor designs have been proposed for rapid, label-free detection of pathogenic bacteria. Lee et al. introduced a standout strategy using multi-resonant microstrip-line-coupled resonators (MMRMs), operating between 0.5 and 10 GHz, to discriminate *Escherichia coli* (*E. coli*) from *Bacillus cereus* at a fixed concentration (~ 0.05 OD₆₀₀/mL) by observing resonant shifts and S₂₁ variations across the first three resonant modes [153]. Piekarz et al. developed a biofunctionalized differential resonant array in Low-Temperature Co-fired Ceramic (LTCC), which is differentially fed and coated with anti-*E. coli* antibodies, achieving a sensitivity of 4.2 Hz·(CFU/mL)⁻¹ and LOD of 10² CFU·mL⁻¹, with no cross-reactivity against *Lactobacillus rhamnosus* [154]. In a related study, the same group fabricated single- and dual-resonant planar biosensors on GaAs (UMS PH25), also functionalized with anti-*E. coli*; they detected concentrations as low as 10³ CFU·mL⁻¹ and established dynamic ranges via calibration curves (SEM confirmation of immobilization; see Fig. 6a) [155].

An integrated microfluidic–microwave biosensor, combining helical resonators and interdigital capacitors, was designed to differentiate *E. coli* from human leukocytes in mixed samples via machine learning (Fig. 6b). The platform combines a network of microstrip resonators with a spiral microfluidic channel on a Rogers 4003 substrate, operating in the frequency range of 0.5 to 3.5 GHz. Classification exploits frequency-response features, particularly 𝑆_11_ shifts, and achieves up to 95.24% accuracy using machine learning algorithms [13]. In contrast, Kumari et al. designed a sensor inspired by electromagnetic neural grid (ENG) structures, featuring CSRR slots for label‑free detection of *Aspergillus niger*, a typical hospital fungal pathogen. Patterned on an FR4 substrate with a central microstrip line, the sensor exhibited a resonant frequency shift from 2.65 to 2.45 GHz when exposed to fungal volatile compounds. An S₂₁ variation of approximately 6.5 dB confirmed the system’s high sensitivity to specific airborne analytes. This design stands out for its simplicity, portability, and suitability for environmental or surface pathogen monitoring [157].

## Microstrip sensors for cellular and tissue characterization

This section focuses on presenting recent advances in cellular- and tissue-level microwave sensing. The conceptual boundary between this section and the preceding one lies in the fundamentally different physical mechanisms involved. Molecular biosensors rely on surface-level perturbations produced by specific biorecognition events, which induce localized resonance shifts near functionalized interfaces. In contrast, the applications reviewed here operate at mesoscopic and macroscopic scales, where the microwave response is governed by volumetric dielectric properties of intact cells, tissues, or multilayer biological phantoms. These responses arise from variations in water content, ionic composition, membrane polarization, cellular density, and tissue morphology, which collectively modulate the propagation and attenuation of electromagnetic fields. For this reason, cellular and tissue characterization constitutes a distinct sensing paradigm that cannot be grouped under molecular biosensing.

### Dielectric detection of cancer cells

Cancer cell lines are produced from human or animal tumors or secretions that, through selection and adaptation, develop the ability to proliferate indefinitely in vitro. Unlike previous approaches that utilize functionalized biosensors to detect specific molecular biomarkers (such as antigens, proteins, or metabolites in biological fluids), microstrip microwave sensors offer an alternative strategy: the direct, label-free dielectric characterization of intact cells or tissues deposited on or flowing across the sensor surface [158]. This method leverages the sensitivity of electromagnetic scattering parameters to global dielectric properties, enabling discrimination between cell types or physiological states without the need for biochemical recognition layers. Differences in resonance frequency, signal attenuation, or phase shifts arise from variations in cellular morphology, internal composition, and functional status, enabling microstrip sensors to distinguish cancer cell lines with high sensitivity [112, 159].

**Fig. 7:**
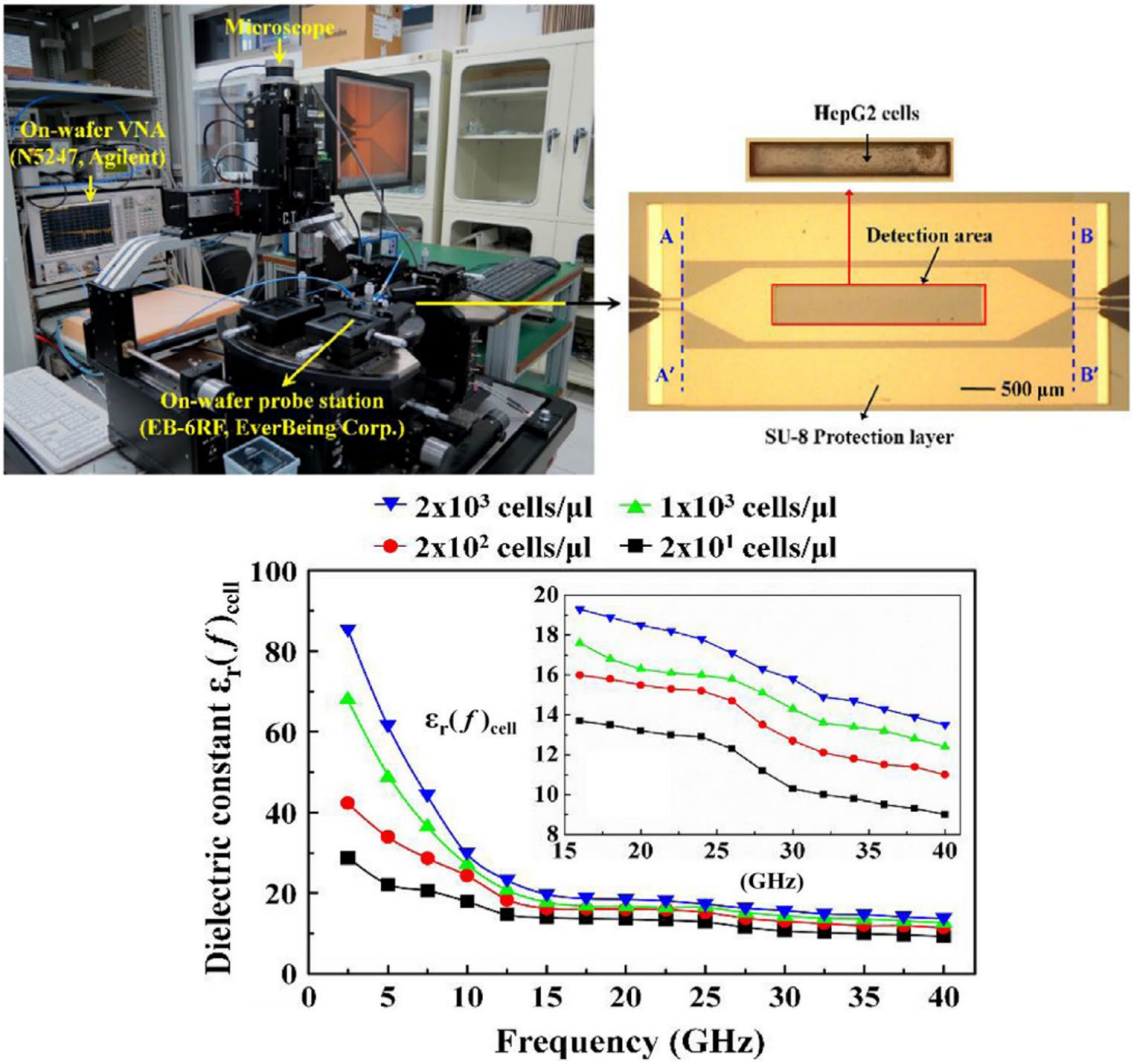
On-chip broadband measurement system for dielectric characterization of hepatoma (HepG2) cells using a CPW biosensor. Scattering parameter measurements up to 40 GHz enabled the extraction of frequency-dependent attenuation and relative permittivity, revealing clear concentration-dependent variations with a sensitivity down to 2 × 10² cells/µL. Adapted with permission from [112]

Microwave biosensor platforms based on microstrip and CPW structures have been extensively investigated for label-free dielectric characterization of living cells. A broadband microstrip biosensor operating from 5 to 40 GHz demonstrated antibody-free differentiation of multiple human cancer cell lines (HEC-1-A, HepG2, A549) [159]. The device enabled the extraction of equivalent electrical models 𝑅cell(𝑓) and 𝐶cell(𝑓) while maintaining sample integrity under ΔT < 4.3 °C with ~ 1 µL volumes, providing reliable wideband analysis with minimal thermal effects. In parallel, a tunable resonant biosensor covering the 1.2–8 GHz range was integrated, featuring a microfluidic chamber, dielectrophoresis electrodes, and a surface-mounted varactor for continuous resonance adjustment [158]. This platform was validated using polystyrene microspheres at a concentration of 5 × 10⁵ particles/mL, where frequency shifts were detected from as few as 12 deposited particles, achieving effective permittivity values of 𝜀_r_ ≤ 2.75. It later demonstrated applicability with glioblastoma cells. Complementarily, a CPW biosensor operating up to 40 GHz enabled accurate dielectric characterization of hepatoma (HepG2) cells [112]. On-chip probe-station measurements yielded frequency-dependent attenuation and cellular permittivity, with detection sensitivity down to 2 × 10² cells/µL. These findings confirm the versatility of microwave biosensing platforms for non-invasive cellular diagnostics, with representative results summarized in Fig. 7.

### Early diagnosis of cutaneous tumors

Early diagnosis of skin tumors, such as basal cell carcinoma, squamous cell carcinoma, and melanoma, is crucial for improving patient outcomes, reducing complication rates, and optimizing therapeutic resources. Although biopsy with histopathological analysis remains the gold standard, it is invasive and unsuitable for large-scale or continuous screening. In this context, electromagnetic sensors offer a complementary approach by exploiting the strong dielectric contrast between malignant and healthy tissues. Melanomas, for instance, can exhibit a 250% increase in relative permittivity and elevated conductivity due to their higher cellular density and water content. Such differences are detectable within millimeter wave and terahertz bands at penetration depths of a few millimeters, making them particularly relevant for non-invasive evaluation of superficial lesions [160].

**Fig. 8:**
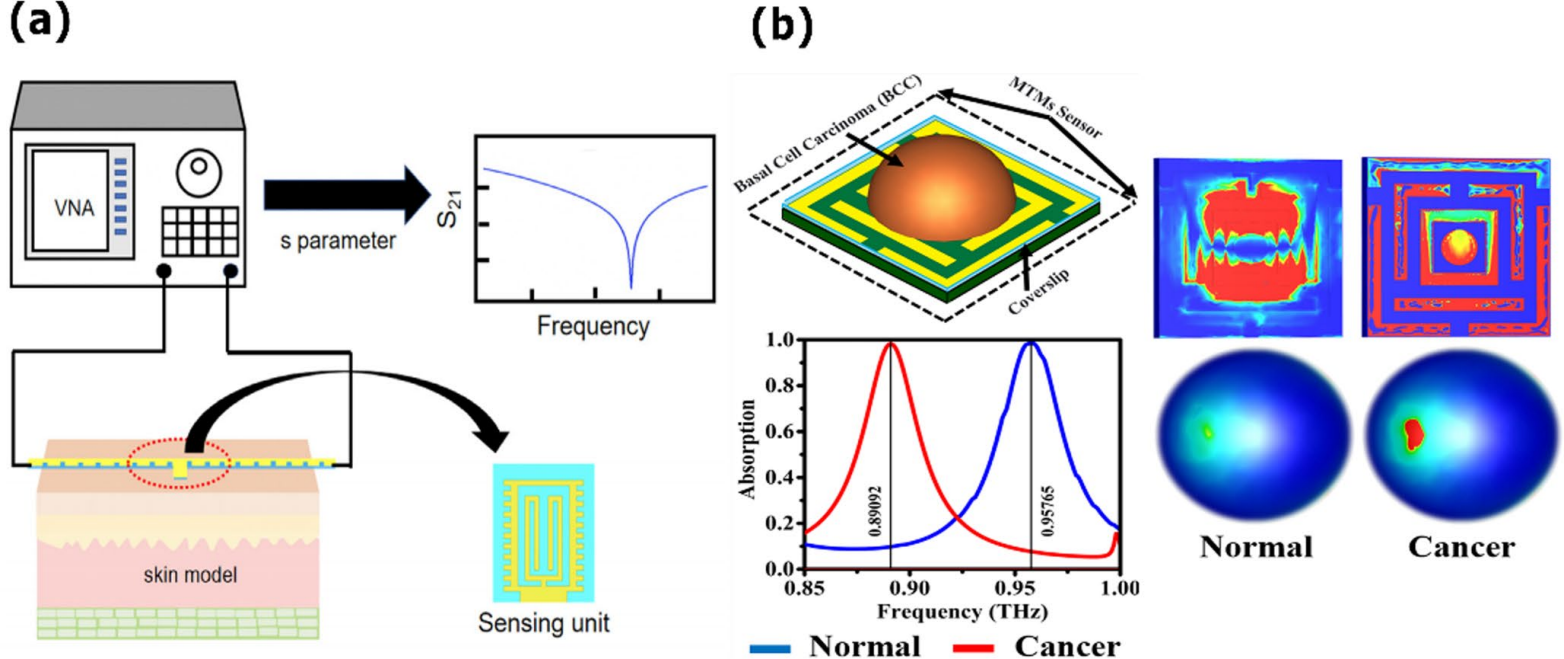
Electromagnetic sensor platforms for non‑invasive cutaneous tumor diagnosis. (**a**) Flexible spoof surface plasmon polariton (SSPP) microstrip sensor conforming to skin curvature for real‑time detection of burns and tumor‑like lesions via S‑parameter analysis. (**b**) Terahertz metamaterial absorber based on a triple‑band perfect‑absorber architecture, developed for highly sensitive dielectric characterization of non-melanoma skin cancers. Adapted with permission from [161, 163] (licensed under CC BY 4.0)

Recent studies illustrate the diversity of sensor strategies targeting this clinical challenge. Arab et al. developed a millimeter wave radar sensor operating at 77 GHz, based on the frequency-modulated continuous-wave principle [133]. This approach enables high-resolution depth profiling by continuously sweeping the carrier frequency, thereby allowing precise localization of subsurface anomalies. The integrated waveguide structure successfully detected simulated melanomas of 4 mm diameter at a depth of 1.5 mm, achieving localization errors below 0.2 mm (Fig. 8a). The method yielded reflection-coefficient contrasts of up to 8 dB, underscoring its potential for contactless lesion screening. Complementarily, Bai et al. designed a flexible spoof surface plasmon polariton sensor fabricated on a thin polymer substrate and operating at 11.3 GHz [161]. The device was tested on ex vivo porcine skin phantoms that simulated burns and hydrated regions. It achieved resonance shifts up to 100 MHz and |S₁₁| variations surpassing 5 dB. The serpentine coupling structure limits the electric field at the surface, resulting in excellent superficial sensitivity suitable for real-time dermal monitoring.

Other groups have focused on wearability and accessibility. For instance, a comparative study by Abd El-Hameed et al. evaluated narrowband and UWB microwave textile sensors for continuous monitoring of vital signs and lung water levels [162]. The device’s evaluation on the breast phantom revealed significant differences in axial ratio and S_11_ between healthy and tumorous tissue, with 12% false positives and 9% false negatives. It also revealed significant differences in the |S11| parameter between various levels of water content, which is used to detect fluid accumulation. While this technique does not offer tomographic imaging, its potential for low cost, mobility, and ease of incorporation into clothes as a chest patch makes it ideal for continuous, non-invasive home monitoring. At higher frequencies, Hamza and collaborators developed a highly sensitive terahertz metamaterial absorber designed exclusively for non-melanoma skin cancer detection [163]. The structure is illustrated in Fig. 8b, which consists of a resonant metamaterial layer inserted between high-permittivity dielectrics, attaining absorption levels greater than 99% at 0.61, 0.88, and 1.19 THz.

### Detection of brain tissue anomalies

Early diagnosis of cerebral abnormalities, such as tumors, intracranial hemorrhages, and ischemic lesions, is crucial for improving clinical outcomes and reducing associated morbidity. While MRI and CT provide high diagnostic resolution, their accessibility is limited by cost, specialized infrastructure, and ionizing radiation exposure [147]. In contrast, microwave‑based sensors offer a non‑invasive, portable alternative capable of detecting dielectric property variations of affected brain tissues. These systems exploit contrasts in relative permittivity and conductivity between healthy parenchyma and pathological regions (e.g., tumor masses or hemorrhages), which manifest as measurable changes in reflection and transmission coefficients. For instance, microstrip patch sensors and UWB antennas have been used to distinguish healthy from atrophic or lesioned brain tissue via shifts in |S₁₁|, central frequency, and signal attenuation in the 0.5–3 GHz range. Such approaches enable real‑time, low‑cost monitoring of structural alterations with clinically sufficient spatial resolution [164]. 

Gugliermino et al. demonstrated a flagship system based on a circular array of 22 monopole patch antennas arranged around a head phantom, operating at a central frequency of 1.1 GHz [164]. When tested on phantoms containing hemorrhagic inclusions, the system achieved 5 mm spatial resolution and detected an average |S₁₁| change of 4.5 dB in the presence of bleeding compared to baseline. Moreover, repeated acquisitions every 5 min tracked the progression of the simulated event, confirming dynamic monitoring capability. Other groups have focused on microwave systems for specific diagnostic applications. For Alzheimer’s disease, Saied and Arslan [165] developed a wearable radio frequency (RF) device composed of six UWB antennas integrated into a hat. This system monitored brain atrophy by detecting changes in the reflection coefficient caused by variations in brain volume and cerebrospinal fluid, a method validated using lamb brain phantoms. For early brain tumor detection, Saleeb et al. [166] introduced a reconfigurable antenna array operating at 2.4 GHz. The detection principle relies on a leftward shift in the reflection coefficient’s frequency response, which correlates to tumor size and enables the identification of tumors as small as 2.5 mm. In the field of stroke monitoring, Rodriguez-Duarte and collaborators [167] designed a portable scanner with a flexible, wearable antenna array for real-time assessment. Their system utilizes an artifact-removal imaging algorithm and was validated using a dynamic phantom that simulates stroke progression, demonstrating high sensitivity in identifying affected areas.

### Other emerging applications: sleep apnea monitoring and inflamed tissues

Monitoring physiological conditions, such as sleep apnea and tissue inflammation, is crucial for the early diagnosis and management of chronic diseases. Conventional diagnostic methods—such as polysomnography and clinical evaluations—are often invasive, expensive, and restricted to hospital settings, which limits their potential for continuous patient monitoring. In contrast, microwave technologies, especially those utilizing microstrip-based sensors, offer a non-invasive and cost-effective alternative that can detect subtle physiological changes. For respiratory monitoring, these sensors can capture movements of the thoracic wall, whereas in inflammation detection, they can measure variations in dielectric properties caused by the presence of excess of fluid. The versatility of microstrip technologies enables diverse sensing architectures, ranging from continuous-wave (CW) Doppler radars [139] and UWB systems [145] for motion detection, to highly sensitive SRRs and complementary CSRRs for characterizing dielectric tissues.

In the field of vital signs monitoring using microwave technology, Baboli et al. demonstrated that a quadrature CW Doppler radar performed comparably to polysomnography, achieving a sensitivity of 86%, a specificity of 91%, and an accuracy of 92% in identifying apnea-hypopnea events [168]. Their automated signal processing revealed a 20% drop in respiration duration during hypopnea and a 40% drop during apnea, establishing radar as a clinically viable option. Pisa et al. further investigated UWB radar for respiratory monitoring, using a detailed thorax model [169]. Their results quantified a 14-ps difference in pulse round-trip time between resting and regular breathing, which increased to 22-ps during deep breathing, confirming UWB’s temporal resolution is sufficient to detect anomalous respiratory patterns.

**Fig. 9:**
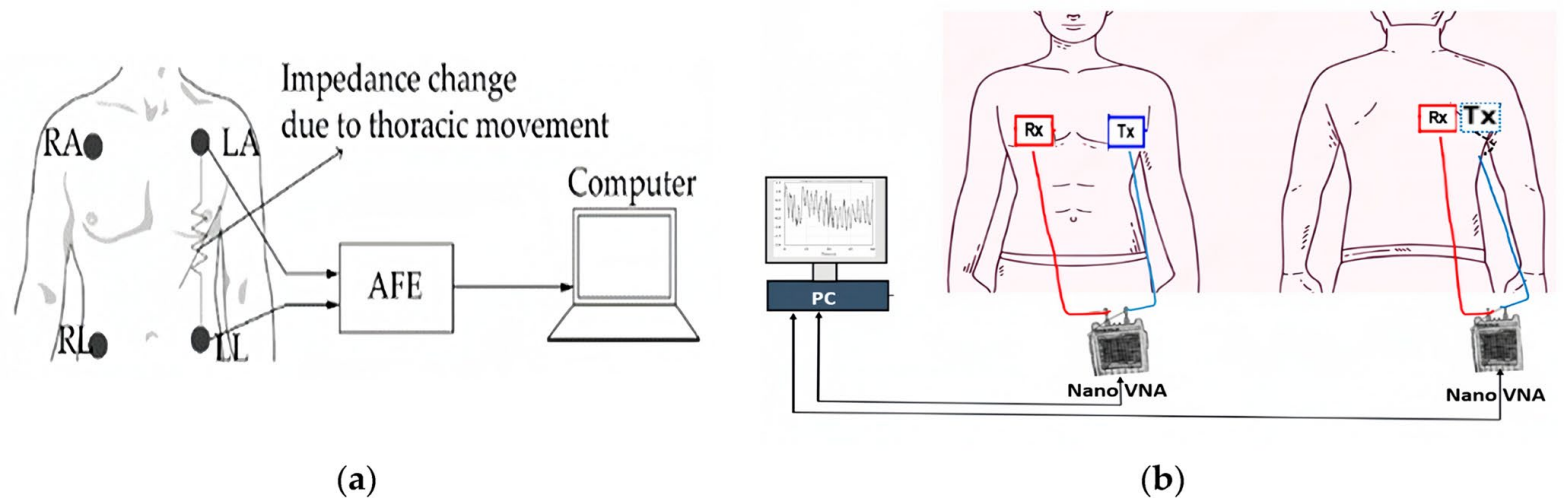
Experimental setups for microwave-based physiological monitoring. **a**) Thoracic impedance monitoring system detecting respiratory motion through electrode-induced impedance changes. (**b**) Dual-transceiver microwave system employing nano vector network analyzers (VNAs) for chest and back measurements, designed to enhance sensitivity to variations in lung water levels associated with edema. Adapted with permission from [162] licensed under CC BY 4.0

In terms of tissue characterization, Abd El-Hameed et al. reviewed microwave sensors designed to accurately measure water levels (LWL), which are vital indicators of edema and tissue inflammation [162]. On a lung phantom with variable water content, a narrowband sensor at 2.4 GHz exhibited a 100 MHz resonance shift and a ~ 2.2 dB variation in |S₁₁| for a 60% increase in water concentration, demonstrating high sensitivity to dielectric changes caused by fluid accumulation. As illustrated in Fig. 9, the experimental setup combined thoracic impedance monitoring with a dual-transceiver VNA configuration, enabling non-invasive assessment of respiratory motion and lung water content.

Emerging fabrication approaches aim to produce low-cost, flexible sensors. Wu et al. developed a paper-based respiratory sensor by screen-printing a multiwall carbon nanotube (MWCNT)/PDMS composite [170]. This device tracks humidity changes during inhalation and exhalation via capacitance shifts, with response times of 1.178 s for increases and 0.88 s for decreases; it successfully distinguishes between normal changes of 150 pF and more profound changes of 350 pF in breathing patterns. A key challenge in these systems is body motion, which can mask physiological signals. To address this, Kiriazi et al., proposed a dual-transceiver cancellation technique in which signals from front and rear transceivers are multiplied, effectively eliminating motion noise up to 5 cm and enabling the reliable extraction of cardiac-beat signals [171].

## Microwave-based medical therapies

The therapeutic use of microwave energy to induce controlled heating in biological tissues has become an essential component of current oncological therapies. This technique is commonly employed in two primary modalities: hyperthermia and microwave ablation (MWA), each with distinct processes and clinical applications. While both involve tissue heating, their therapeutic approaches differ. Hyperthermia exposes tumors to moderate temperatures (40–45 °C) for extended periods (60–90 min), not immediately killing cells, but sensitizing them to radiotherapy and chemotherapy by producing sublethal stress [33, 149]. In contrast, MWA creates much higher temperatures (> 50–55 °C), resulting in irreversible protein denaturation and coagulative necrosis, with the explicit purpose of eradicating tumors [32, 172].

The physical basis of both modalities stems from the interaction of oscillating microwave fields with tissue water dipoles. This energy transfer is quantified by the SAR, while heat diffusion and perfusion dynamics can be modeled using Pennes’ bioheat equation [173]:

$$\:\rho\:c\frac{\partial\:T}{\partial\:t}=\nabla\:(k\nabla\:T)+{\rho\:}_bc_b{\omega\:}_b(T_b-T)+Q_{MW}$$

where $\:\rho\:,\:c\:and\:k$ are tissue density, specific heat, and thermal conductivity; $\:{\rho\:}_{b}{c}_{b}\:and\:{\omega\:}_{b}$ are blood density, blood specific heat, and perfusion rate, $\:{T}_{b}$, is blood temperature and $\:{Q}_{MW}$ is the microwave‑induced heat source.

### Microwave ablation: principles, clinical applications, and theranostic advances

**Fig. 10:**
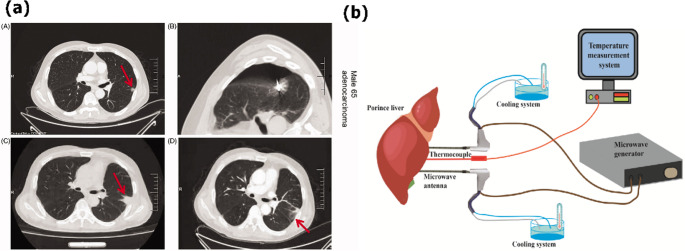
Principles, technology, and clinical application of microwave ablation (MWA). (**a**) Physical principle: microwave electromagnetic fields induce oscillation of water molecules in tissue, generating frictional heat and coagulative necrosis. (**b**) Clinical outcome: CT images of a lung lesion (adenocarcinoma) before treatment, during applicator insertion, and post‑MWA showing a ground‑glass opacity zone indicative of successful ablation. Adapted from [172, 174]. Licensed under CC BY 4.0

Clinical and preclinical studies have consistently demonstrated the efficacy of MWA compared to other ablative approaches. In a propensity-matched study, Xu et al. reported that MWA combined with chemotherapy achieved superior long-term outcomes in lung cancer patients compared to radiofrequency ablation (RFA) due to local tumor control rates reaching 90.6% for MWA versus 78.1% for RFA, and progression-free survival was extended to 10.4 months (vs. 9.2 months), with comparable complication rates (see Fig. 10a) [174]. Preclinical validation by Brace et al. using a 17G triaxial antenna in porcine liver models confirmed the ability of MWA to produce uniform, near-spherical ablation zones (> 2 cm diameter) with low reflected power (< 4%) even under perfused conditions [175]. On the technological side, Atigh et al. numerically investigated slotted coaxial antennas in combination with phase-change materials to mitigate collateral damage to healthy tissues [172, 174, 176]. Their simulations demonstrated that PCM injections reduced surrounding tissue temperatures by ~ 5 °C while maintaining effective tumor heating, highlighting a novel strategy for thermal protection and enhanced ablation precision.

Improvements in image-guided workflows have further optimized clinical use. Du and collaborators demonstrated that CT with a C-shaped fluoroscopy system (C-arm CT) guidance during pulmonary MWA reduced total procedure and needle placement times, while also decreased radiation dose, without compromising survival outcomes compared to conventional CT [177]. Moreover, synergistic protocols are being explored. For example, Li et al. reported that combining MWA with apatinib (a VEGFR-2 inhibitor) and camrelizumab (an anti-PD-1 antibody) in advanced hepatocellular carcinoma patients resulted in encouraging response rates and a median overall survival of 19.3 months, highlighting the potential of multimodal strategies that integrate local ablation with systemic therapies [178]. Complementarily, Wang et al. developed and validated a dual-antenna MWA platform (see Fig. 10b), using finite element simulations and ex vivo porcine liver experiments to demonstrate that closely spaced applicators (20–30 mm) produce larger, more uniform coagulation zones suitable for tumors exceeding 5 cm [172, 174, 176]. Their results also highlighted the influence of blood perfusion on ablation volume, underscoring the importance of precise applicator design and spacing in clinical translation.

These advances have been translated into broader clinical adoption. Today, MWA is a standard of care in hepatic oncology, particularly for hepatocellular carcinoma and colorectal metastases, where it achieves lower local recurrence than RFA [179]. Its indications have expanded to pulmonary and thyroid malignancies, with high technical success and favorable safety profiles [160, 180]. Comparative analyses further underline that MWA is less affected by the heat-sink effect of adjacent vessels and is more effective in tumors ≥ 3 cm [181]. Yet, as Marcelin et al. demonstrated, in vivo ablation zones may differ significantly from ex vivo predictions, reinforcing the need for accurate intraoperative monitoring [181]. To guide clinical practice, Cai et al. proposed preclinical guidelines recommending moderate power (< 60 W) and short durations (< 8 min) to achieve predictable ablation volumes in lung tissue [182].

Looking ahead, MWA is evolving beyond a purely destructive technique toward integrated therapeutic platforms. Theranostic approaches are particularly promising, as they combine energy delivery with diagnostic or monitoring capabilities. Hyperthermia, which involves moderate heating, has also been the subject of intensive optimization. For example, Acar et al. presented a two-step optimization technique for reducing hot patches in breast phantoms while focusing heat within the tumor [183]. Similarly, Firuzalizadeh et al. addressed the issue of inadequate antenna matching in head and neck treatments, demonstrating how simultaneous optimization of focusing and power transmission preserved efficiency without compromising safety [184]. At the prototype level, Stang et al. developed a preclinical system that employs time-reversal approaches based on prior imaging to focus microwave energy. Using a 12-element tapered microstrip patch array operating at 915 MHz, the system achieved well-defined focal spots of approximately 1.5 cm in breast-mimicking phantoms, with significant differential heating confined to the target region. Beyond proving the feasibility of precise focal control, this study also underscored the potential for integrating patient-specific imaging data into multiphysics models and highlighted avenues for real-time thermal monitoring (e.g., ultrasound tomography or microwave radiometry), reinforcing the theranostic potential of such approaches [185].

Finally, microwave technologies are being utilized for therapy monitoring, paving the way for theranostic treatments. Golnabi et al. created a microwave imaging system capable of reconstructing tissue dielectric characteristics into 2D and 3D maps, which was confirmed by following a breast cancer patient during 135 days of neoadjuvant therapy [186]. The system not only detected tumor regression in accordance with radiological findings, but it also demonstrated microwave imaging’s promise as a safe, functional, and non-ionizing technique for assessing treatment efficacy in real time.

### Fundamentals of microwave hyperthermia (MH)

Microwave hyperthermia relies on the interaction of frequency-dependent penetration depth, applicator–tissue coupling, and energy-focusing techniques, which heat cancerous tissue while leaving healthy tissue nearby unaffected. Lower frequencies, such as 434 or 915 MHz, penetrate deeper, making them suitable for visceral and muscle tumors. Higher frequencies, like 2.45 GHz, concentrate energy on the surface, which is suitable for skin and breast lesions [72, 187–189]. In this context, computational models have demonstrated that malignant tissue absorbs microwave energy more effectively than healthy tissue, particularly when temperature-dependent dielectric properties are integrated into bioheat simulations. These studies indicate that low frequencies (around 915 MHz) are most effective for treating deep tumors, as they distribute heat more evenly. Higher frequencies (above 2.45 GHz) are more effective in controlling the heating of surface targets. Changing the frequency, power, and exposure time can reliably raise tumors into the therapeutic window while keeping the skin around them at about 40–41 °C [190].

On the other hand, some improvements in applicator design have made MH more accurate (Abdel-Haleem et al. [191]). Altintas et al. created a linear Vivaldi array at 915 MHz MH [192]. Their approach reduced computational complexity compared to conventional optimization algorithms, making adaptive, patient-specific planning more feasible. Similarly, Kaur et al. proposed an artificial magnetic conductor (AMC)-backed Archimedean spiral antenna at 2.5 GHz for superficial cancers [193]. This antenna enhanced gain and directivity, creating consistent SAR distributions and reaching temperatures of 43–45 °C in both superficial and sub-surface tumors with little input power. Compact folded-dipole applicators at 2.45 GHz further demonstrated regulated heating footprints suited for melanoma therapy, without the use of water boluses [188].

**Fig. 11:**
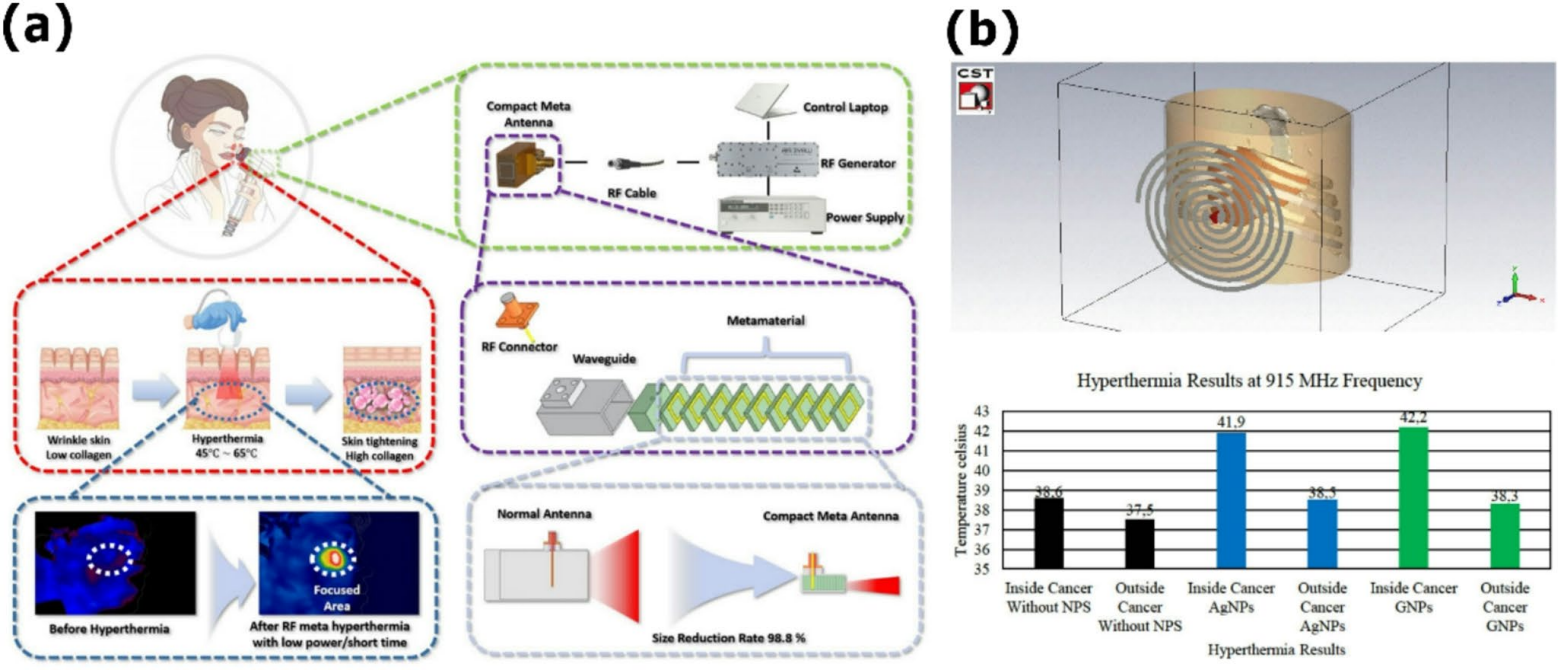
Advanced techniques in Radio Frequency (RF) and Microwave Hyperthermia (MH). (**a**) RF hyperthermia system for cosmetic skin tightening. A compact metamaterial-filled antenna focuses RF energy on the dermal layer, stimulating collagen to achieve a skin-tightening effect. (**b**) Nanoparticle-enhanced MH for cancer therapy. A spiral antenna treats a liver tumor, with gold nanoparticles (GNPs) acting as microwave absorbers to selectively increase the tumor’s temperature to a therapeutic level (42.2 °C) while sparing healthy tissue. Adapted from [195, 196] Licensed under CC BY 4.0

In addition to applicator design, nanoparticle-assisted methods have been shown to enhance therapeutic ratios significantly. Petryk et al. found that magnetic nanoparticle hyperthermia (mNPH) produced more homogenous necrosis and lower peritumoral damage than conventional 915 MHz hyperthermia, even when equal heat doses were administered [194]. This evidence suggested that nanoparticle-assisted heating can overcome one of the main limitations of MH—heterogeneous temperature distribution. McWilliams et al. found that spherical Fe/Fe₃O₄ nanoparticles, especially those around 10 nm in size, improved microwave-induced heating and increased the effective treatment zone in tissue phantoms [187]. Their work also clarified that the electric field component dominated the enhancement mechanism, providing valuable guidance for applicator design. Kim et al. developed a small metamaterial-filled waveguide aperture antenna that operates at 2.45 GHz for cosmetic skin tightening [195]thereby expanding the use of MH beyond oncology. The device, with an aperture of only 10 × 17.4 mm, achieved highly focused near-field heating, elevating skin temperatures by 11.6 and 35.3 °C, respectively, using 20 and 80 W of input power, in less than 60 s. Figure 11a illustrates a schematic of this RF system, showing how metamaterials enable downsizing while enhancing heating efficiency. In liver cancer models, Maamoun et al. used a planar Archimedean spiral antenna at 915 MHz and demonstrated that the integration of gold nanoparticles increased SAR by more than 30-fold and allowed tumors to reach therapeutic temperatures (> 42 °C) without harming surrounding healthy tissue [196]. Figure 11b illustrates the proposed arrangement, emphasizing the importance of antenna layout for selective and localized hyperthermia administration. These results underscore the potential of nanoparticle-assisted MH to selectively boost energy absorption at the tumor site, improve homogeneity of heating, and expand the clinical applicability of hyperthermia to tumors that are otherwise difficult to treat with conventional applicators alone.

## Integration of advanced functionalities in microwave (bio)sensors

Microwave biosensing has advanced from proof-of-concept devices to multipurpose platforms that integrate clinical usefulness, miniaturization, and sensitivity. To achieve this, scientists are combining complementary technologies that enhance the functionality and performance of microwave sensors. These developments extend beyond enhancing detection limits; they also address essential requirements in everyday and clinical contexts, such as portability, intelligent data interpretation, and continuous monitoring.

The next generation of microwave-based biomedical devices is being shaped by four main directions, which are reviewed in this section. First, direct interaction between cells and biomolecules, as well as the electromagnetic field, is made possible by microfluidic integration, which also enables the accurate handling of volumes at the microliter scale. Second, the range of microwave sensing from labs to POC is being expanded by the development of wearable, implantable, and portable sensors that allow for continuous and minimally invasive monitoring. Third, the application of ML to predictive data analysis is enhancing specificity and facilitating early detection in complex biomedical contexts by converting unprocessed electromagnetic signals into actionable diagnostic data. Lastly, the groundwork for IoMT ecosystems is being laid by multiparametric and networked sensing platforms.

Microfluidics, wearable and implantable architectures, machine-learning–assisted analysis, and multiparametric or IoMT-connected platforms do not constitute independent diagnostic modalities. Rather, these functionalities act as cross-cutting enablers that enhance sensitivity, stability, portability, automation, and translational scalability across both molecular biosensing and cellular- and tissue-level dielectric characterization. For this reason, these functionalities are presented in a dedicated section emphasizing their transversal role and their unifying impact on the clinical readiness and technological maturity of microwave biosensing platforms.

### Microfluidic integration in microwave (bio)sensors

Channeling microliter volumes through pre-defined microchannels over the (bio)sensor’s active region enhances the dielectric interaction between the sample and the electric field, which is one of the benefits of increasing automation, miniaturization, and sensitivity. This convergence enables effective and repeatable interaction by providing control over the exposure, confinement, and transportation of small amounts of biological samples. Well-placed channels maximize the sample-field dielectric coupling instead of geometrically “delimiting” the active volume; for instance, putting the channel exactly where the line/resonator’s field is maximum increases sensitivity, decreases diffusion, evaporation, and cross-contamination artifacts. It is observed in microstrip architectures integrated with microchannels in PDMS or porous membranes, which operate with volumes of a few microliters and exploit the field distribution for their characterization of fluids and cells [133, 197]. In practice, this integration must also contend with biofouling, channel clogging, and material compatibility with reagents and cleaning protocols, which are critical for repeated measurements and long-term stability in clinical workflows.

To achieve reliable detections in complex biological media, biochemical functionalization in microstrip (bio)sensors, combined with microfluidics, is essential. Bioreceptors can be immobilized by internally treating the microchannel through oxidation, silanization, or coating with adhesive layers during the fabrication process. These steps can be carried out in situ or before assembly by adding biomolecules and activating solutions under controlled flow conditions. To immobilize the bioreceptors precisely in the region of maximum coupling and prevent interference with the electromagnetic response, the design should expose the functionalized sites to the (bo)sensor’s evanescent field while preserving nL-µL volumes and avoiding turbulence [197, 198].

Recent experimental work demonstrates the adaptability of these strategies. Amanati Shahri et al. presented a λ/4 stub microstrip sensor aligned with a nanowire-filled microchannel [133]. As cells move through the channel, they pass directly over the region of maximal electric field, resulting in noticeable phase alterations in the S_21_ parameter. For a single cell, the mean variation is 22.85 ± 1.65° at ~ 11.1 GHz, indicating label-free detection of individual events. The authors describe a micromachining technique involving sputtering, laser lithography, and etching (channels) with PDMS assembly and a 50 Ω CPW carrier for reliable measurement without RF spikes.

Expanding the scope of integration, Li et al. proposed a hybrid microwave-microfluidic technology that integrates inertial spiral microfluidics for mechanical separation with on-chip dielectric measurement [199]. The system successfully separated 85.3% of leukocytes and yeast with sensitivities of 2.53 MHz·(10⁵ mL⁻¹)⁻¹ and 145.36 pF·(10⁵ mL⁻¹)⁻¹, respectively. These findings directly relate changes in effective permittivity to quantifiable resonance shifts. Grenier et al. developed the methodological foundation for this field by combining broadband microwave interrogation (0.4–35 GHz) and microfluidics [197]. The transmission-line model and de-embedding strategy enabled the accurate dielectric characterization of cellular suspensions with as little as 3 µL of sample, reporting an effective relative-permittivity decrease approaching a factor of two (≈101% shift at 20 GHz) and reproducibility close to 1% above 20 GHz..

**Fig. 12:**
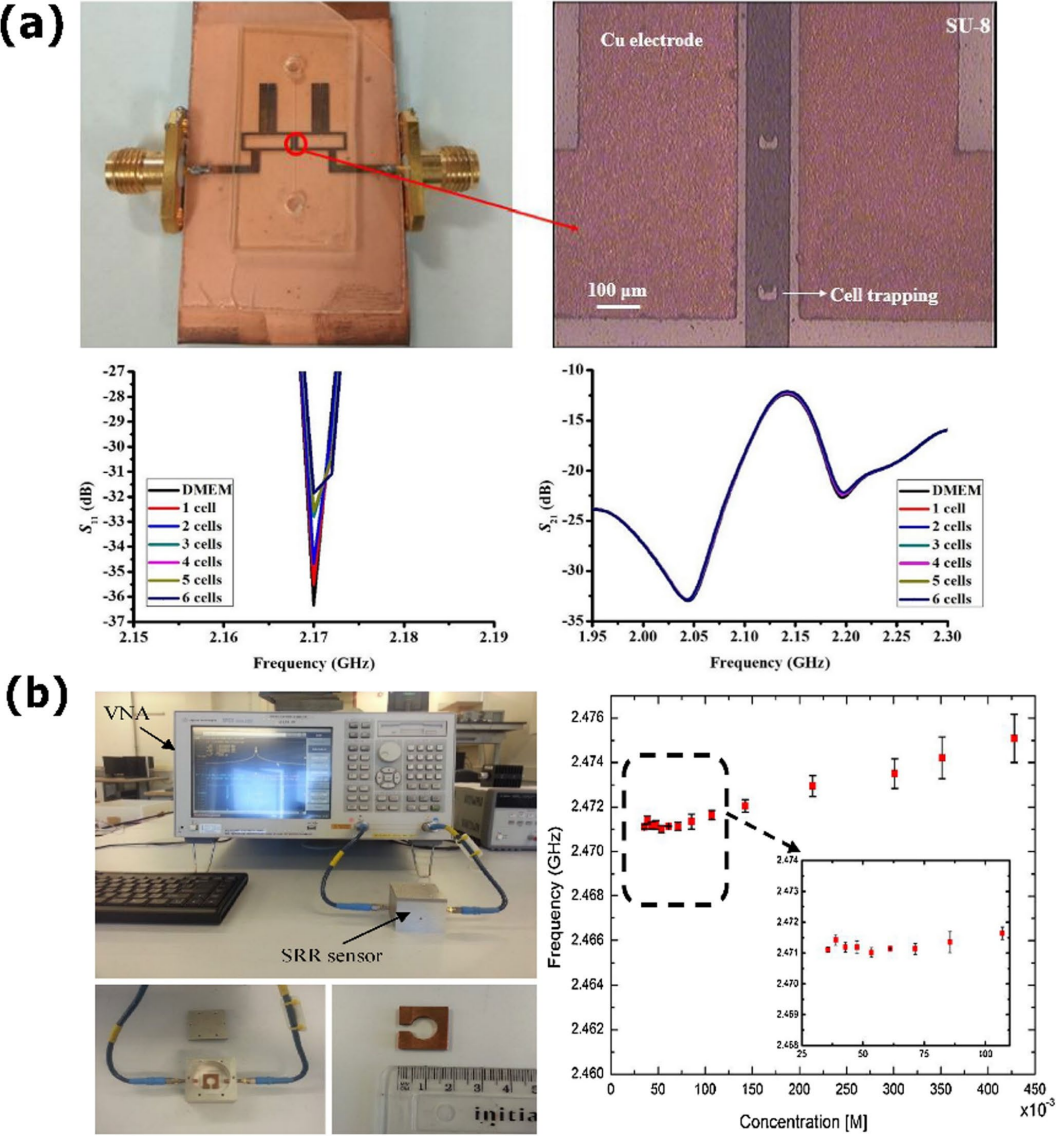
Integration of advanced functionalities in microwave–based microfluidic sensors. (**a**) Single-cell detection technology, where microfluidic channels precisely guide individual cells into the active region of a fork resonator. Each cell induces a measurable shift in the microwave response, allowing for a quantitative correlation with cell count. (**b**) Optimized sensitivity principle, in which the orientation of a SRR relative to the microfluidic channel maximizes electric field interaction and minimizes depolarization. This configuration enables the detection of subtle ionic variations in the fluid as changes in resonator energy dissipation, which is quantified through its Q-factor. Adapted with permission from [200, 201]

Beyond proof-of-concept devices, researchers have demonstrated applications with direct biomedical relevance. For example, Liu et al. developed a fork-resonator sensor operating at 2.17 GHz for label-free, quantitative single-cell counting [181] (Fig. 12a). The system produced a linear S_11_ response with a sensitivity of 0.7 dB per cell, laying the groundwork for microwave cytometry as a non-invasive method for probing the biophysical properties of cells. In parallel, Hamzah et al. advanced liquid analysis by addressing one of the key limitations of aqueous sample signal depolarization [200]. By aligning the fluid channel parallel to the electric field (see Fig. 12b), they maximize the quality factor (Q-factor) response of a SRR. This orientation enabled highly accurate, label-free monitoring of ionic properties, achieving pH measurements with an error of less than ± 0.01 and precise quantification of salt concentration.

Taken together, these examples illustrate that lab-on-chip microwave platforms are not only miniaturized copies of bulk systems, but architectures in which fluid dynamics, surface chemistry, and resonant field distribution must be co-designed. Outstanding challenges include standardizing microfluidic–RF interfaces, ensuring manufacturability at scale, and integrating on-chip actuation (pumps, valves) and sample preparation steps, which are essential for moving from controlled laboratory demonstrations to robust point-of-care diagnostics.

### Development of portable, implantable, and wearable sensors

The development of microstrip sensors with wearable, implantable, and portable capabilities is one of the most active areas in the field. These sensors demonstrate outstanding potential for continuous health monitoring and personalized medicine. However, sensitivity alone is no longer sufficient in this case. Additionally, sensors need to be energy-efficient, biocompatible, ergonomic, and easily integrated with wireless transmission technologies, such as energy-harvesting rectennas, Bluetooth Low Energy (BLE), and passive Radio Frequency Identification (RFID). Microstrip architectures, which integrate sensing, energy sources, and data transmission into textiles, skin-mounted devices, and even implantable structures, are becoming increasingly essential to sustainable wearable systems, according to recent investigations, such as those by Ah et al. [62]. Similarly, Chiao et al. highlighted that microwave-based implantable platforms not only enable precise sensing but also facilitate intra- and inter-body communication, opening new possibilities for wireless diagnostics and therapy [29]. In wearable operation, these devices must also tolerate body motion, tissue-induced detuning, and strict SAR limits, which impose additional constraints on antenna geometry, matching networks, and operating frequency.

**Fig. 13:**
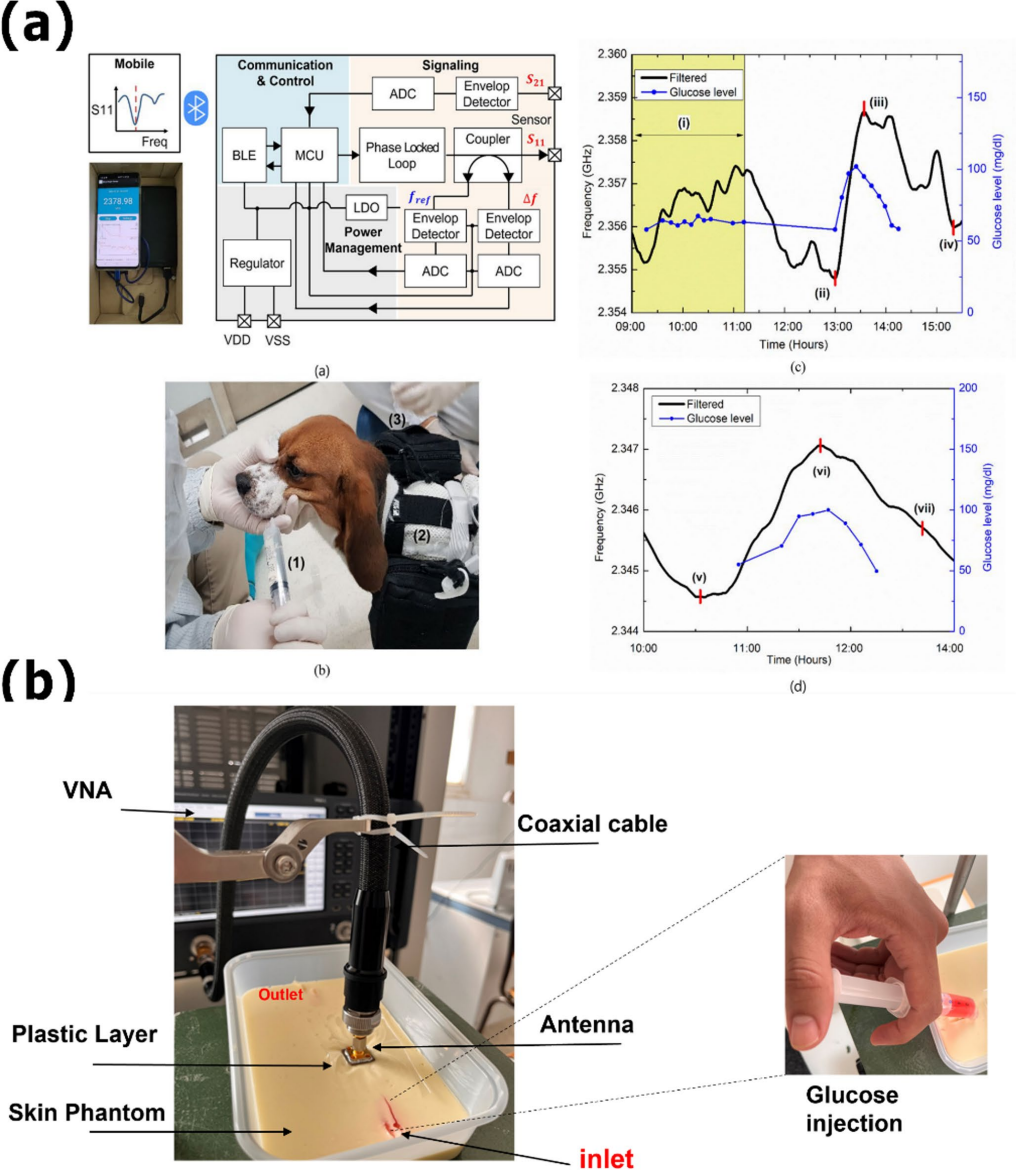
Platforms, validation methods, and key components in microwave biomedical sensors. (**a**) An implantable electromagnetic biosensor is connected to a portable interface unit with Bluetooth communication, enabling continuous glucose monitoring in a beagle model. The recorded data shows a strong correlation between the sensor’s resonance frequency and blood glucose levels over several hours. (**b**) An experimental setup using a skin-mimicking phantom with embedded venous channels to test the response of a microwave antenna. Glucose solutions at different concentrations are injected to simulate physiological changes under controlled laboratory conditions. Adapted from [203, 205] licensed under CC BY 4.0

Recent developments have demonstrated progress in this area. In their review of the state-of-the-art microwave sensors for glucose monitoring, Kandwal et al. noted enduring difficulties, including detecting minute changes in blood permittivity, ensuring specificity against other metabolites, and achieving proper miniaturization [202]. In fact, compact radar-like modules or integrated circuitry that allow true portability are replacing large VNAs to address these issues. Furthermore, Kim et al. demonstrated a subcutaneous electromagnetic biosensor for continuous glucose monitoring, which was verified in beagles and pigs, as illustrated in Fig. 13a [203]. In that case, the cylindrical sensor (15 mm × 4 mm) measured dielectric variations in interstitial fluid correlated with blood glucose, achieving a sensitivity of 104 kHz·(mg/dL) ⁻¹ and a mean absolute relative difference (MARD) of 9.67%. Data were transmitted via a portable Bluetooth interface and visualized in real time through a mobile application.

Additional implantable solutions concentrate on enhancing miniaturization and signal stability. A wireless implantable system optimized for intra-organ communication was designed by James et al. using a microstrip antenna loaded with CSRR [204]. With losses of −10 dB and a 38% size reduction compared to conventional antennas, the device transmitted steadily at 2.45 GHz through up to 30 mm of simulated tissue. In addition, Zaarour et al. used a semi-solid skin phantom with embedded channels that mimicked superficial veins to emulate physiological glucose variations under controlled conditions and evaluate the antenna response[205]. The mimicked model is shown in Fig. 13b. Their 14 GHz miniaturized microstrip antenna, tuned for shallow penetration (~ 1.5 mm), demonstrated high linearity for non-invasive glucose detection with a sensitivity of 0.41 MHz·(mg/dL) ⁻¹. It also correlated resonance frequency shifts with glucose concentration (60–200 mg/dL).

Finally, a small, broadband, implantable antenna that can function reliably in the intricate dielectric environment of the human body was introduced by Gan et al. [206]. The antenna was encased in silicone for biocompatibility and had a small size (6 × 6 × 0.53 mm³), which enabled it to achieve a 28% relative bandwidth (2.3–3.03 GHz), allowing it to cover the industrial, scientific, and medical (ISM) band fully. One of the safest and most effective designs to date, tests in pig tissue showed mechanical robustness, stable omnidirectional radiation, and incredibly low SAR values (0.0111 W/kg for 1 g of tissue). These results underscore that wearable and implantable microwave sensors must simultaneously address RF performance, long-term encapsulation, mechanical compliance with soft tissues, and seamless integration with off-body receivers, making them intrinsically multiphysics design problems that extend beyond traditional antenna engineering.

### Machine learning and artificial intelligence–assisted microwave sensing and therapy

The growing volume of data generated by microstrip sensors—particularly under dynamic or variable physiological conditions—has accelerated the integration of ML as a powerful tool for predictive analysis. By extracting complex nonlinear patterns, ML models enhance diagnostic sensitivity and help compensate for uncontrolled variations such as temperature drifts, motion artifacts, or electromagnetic interference. In fact, Seyyedmasoumian et al. described in a recent review how ML can be applied throughout the dielectric sensing workflow, from the structural design to advanced post-processing for material classification and environmental compensation [207]. Similarly, Santra et al. emphasized the broader role of AI across RF sensors, demonstrating how deep learning methods caneplace conventional signal-processing approaches to improve robustness, accuracy, and resilience in biomedical monitoring [208]. In biosensing applications, ML has been deployed both as a regression engine that maps resonant features to continuous biophysical parameters, such as permittivity, analyte concentration, or cellular density, and as a classifier that distinguishes tissue types, disease states, or mixture compositions with accuracy surpassing manually engineered metrics based solely on frequency shift or amplitude variation.

The usefulness of ML integration has been demonstrated by experimental research. Lei et al. created a microwave biosensor based on an SRR that operates at 4.33 GHz to detect carcinoembryonic antigen [134]. The system demonstrated the potential of hybrid hardware–software platforms for ultra-sensitive biomarker monitoring, achieving excellent linearity (R² = 0.999), high sensitivity (27.5 MHz/(ng/mL)), and a low detection limit of 39 pg/mL when combined with a convolutional neural network (CNN). Additionally, Patel et al. investigated AI-assisted structural optimization to develop a graphene metasurface biosensor for hemoglobin detection [209]. They attained a phenomenal sensitivity of 2000 nm per refractive index unit (RIU) by tuning geometrical parameters using polynomial regression models, demonstrating how ML model accelerates sensor design and performance prediction. Kazemi et al. presented an SRR-based sensor operating at 1.19 GHz, paired with support vector machines (SVMs), to categorize acetone/methanol mixtures independently of ambient temperature and address the issue of thermal drift [210]. Their method obtained 99.4% classification accuracy, demonstrating ML’s ability to extract relevant biological or chemical information from environmental perturbations.

In addition, recent literature demonstrates that coupling microwave microstrip and microfluidic sensors with data-driven and ML frameworks significantly enhances performance and translational potential. For example, a 2025 review on microwave medical imaging (MMI) reports that ML and deep-learning algorithms applied to MMI have yielded substantially improved outcomes in lesion detection, tissue classification, and image reconstruction, outperforming traditional inverse-scattering and classical reconstruction techniques in terms of accuracy, spatial resolution, and computational efficiency [211].

Another compelling example is from AI-assisted ultra-high-sensitivity/resolution active-coupled CSRR-based sensor with embedded selectivity (2023), where a planar microwave sensor based on coupled cCSRRs at 2.45 GHz was used for liquid mixture sensing. By incorporating a regenerative amplifier to boost the quality factor (from Q ≈ 70 to 2700) and applying a deep neural network (DNN) on features such as frequency shift, amplitude, and Q-factor, the authors significantly enhanced selectivity and sensitivity. For binary mixtures, concentration error was under 4.3%; for more complex mixtures (ternary), use of a CNN on the full transmission response reduced maximum error to ≈ 0.7% — a roughly sixfold improvement over traditional analysis [212]. A 2025 study Design of AI-driven microwave imaging for lung tumor detection proposed a wearable-antenna MWI system (eight-antenna belt) combined with ML models — in this case a classifier based on XGBoost on raw S-parameter data, and a CNN on reconstructed images — to detect lung tumors and estimate tumor size. The authors reported that the XGBoost classifier outperformed the CNN for tumor detection, while the CNN performed better for size estimation, highlighting that different ML approaches may suit different diagnostic aims, and that raw parameter-based ML may rival image-based ML for certain tasks [213].

**Fig. 14:**
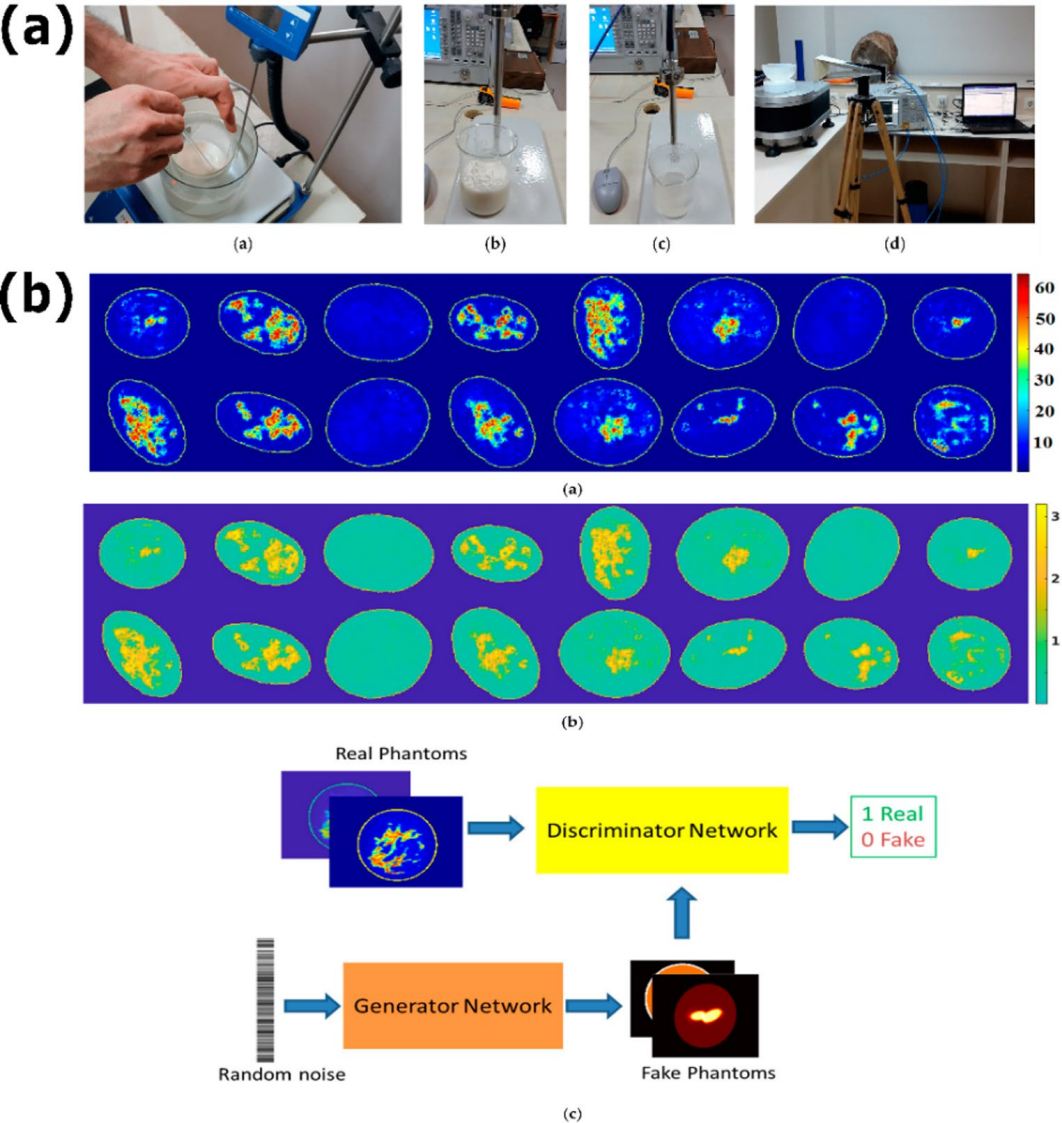
Representative examples of ML–assisted microwave imaging workflows used in biomedical applications. (**a**) Phantom fabrication, characterization, and experimental measurement procedures employed for validating microwave imaging systems, illustrating healthy and tumorous dielectric phantoms and the corresponding measurement setup. (**b**) GAN framework for dielectric breast phantom generation, showing synthesized relative permittivity and conductivity maps and the GAN training architecture. Adapted from [211], licensed under CC BY 4.0

In a recent comprehensive review, Shao systematically analyzed the impact of ML on microwave biomedical imaging using both experimental and clinically validated data [211]. As illustrated in Fig. 14a, physically fabricated healthy and tumorous dielectric phantoms are used to generate reliable experimental datasets for validating microwave imaging systems. Building upon such data, Shao reported that SVM classifiers applied directly to measured microwave responses achieved 91% accuracy, 84.4% sensitivity, and 95.5% specificity in breast tumor detection, demonstrating the effectiveness of ML models operating on raw electromagnetic measurements. Furthermore, the review highlights how data scarcity and computational cost can be mitigated through data-driven approaches. As shown in Fig. 14b, generative adversarial networks (GANs) have been employed to synthesize realistic dielectric breast phantoms by learning the statistical distributions of relative permittivity and conductivity, enabling large-scale dataset generation and improving the robustness and generalization of ML-assisted microwave imaging frameworks.

Overall, the integration of ML and AI methodologies into microstrip-based sensors, resonant platforms, and microwave imaging systems not only improves sensitivity, selectivity, and robustness, but also enables real-time decision making, adaptive calibration, and automated feature extraction across biomedical applications. By linking hardware-level electromagnetic transduction with data-driven inference, these hybrid architectures represent a key enabling technology for next-generation microwave diagnostics and minimally invasive therapies, reinforcing their role within the broader category of Advanced Functionalities.

### Multiparametric and networked (bio)sensing platforms for wearable and IoMT applications

The development of multiparametric detection platforms connected to wireless networks represents one of the most notable developments in the field of biosensors applied to personalized medicine and continuous physiological monitoring. These architectures aim to simultaneously quantify multiple biological variables, such as heart rate, temperature, or respiratory rate, in dynamic environments without requiring user intervention. Integration with communication technologies, such as BLE, Wi-Fi, or RFID, allows for coupling with remote clinical infrastructures, enabling IoMT schemes. Compared to single-parameter devices, microwave multiparametric platforms can exploit the frequency, amplitude, phase, and polarization dimensions of the signal to encode several physiological variables in parallel, while wireless links provide both data transfer and, in some cases, remote powering.

Recent developments in IoMT devices represent a step forward toward increasingly miniaturized, sensitive, and biocompatible platforms. For example, there are reports of a flexible and biodegradable implantable antenna, made of mangosteen biopolymer, which has a small size of 13 × 10 × 1.07 mm³. The sensor device achieves a bandwidth of 565 MHz (ISM 2.4–2.48 GHz) and 2.12 GHz (Sub6 5G), operating with safe powers of up to 2.3 mW and a transmission capacity of 2 Mbps at 5 m [214]. At the same time, a double-layer metamaterial resonator for dengue detection demonstrated high sensitivity, with a shift of 0.325 GHz per unit of blood permittivity, achieving high repeatability suitable for networked biosensing [215].

Parallel progress is seen in skintronics, which combine ultrathin form with biocompatibility and self-healing capabilities. These systems utilize highly conductive materials and transition metal carbides, nitrides, or carbonitrides (MXene)-based structures with exceptionally high gauge factors, allowing for continuous monitoring of physiological signals while maintaining comfort and providing seamless wireless connectivity via Bluetooth or NFC [216]. Iqbal et al. developed a flexible multimodal antenna-sensor capable of simultaneously monitoring three physical parameters (deformation, temperature, and humidity) [217]. This sensor operates within the 2.45 GHz ISM band. The device achieved sensitivities of 23 MHz/mm for deformation, 15.7 MHz/°C for temperature, and 1 MHz/%RH for humidity, all with a single resonant structure. Such multimodal platforms illustrate how a single microwave resonant element can be engineered to decouple or jointly encode several stimuli, enabling richer physiological profiling without increasing device footprint.

**Fig. 15:**
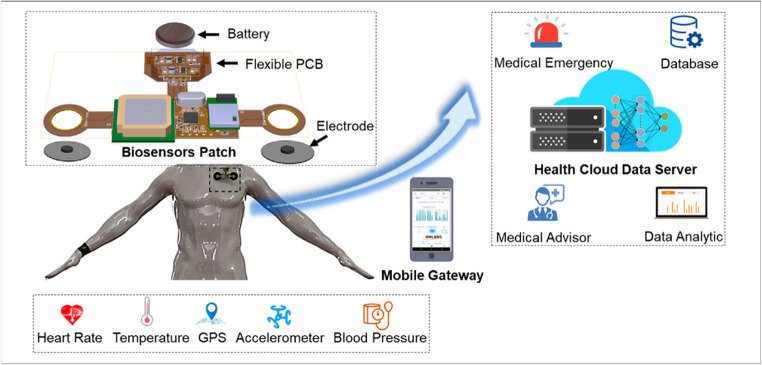
Schematic illustration of a multiparametric wearable biosensing platform integrated within an Internet of Medical Things (IoMT) architecture, showing the acquisition of multiple physiological signals at the wearable level, wireless data transmission to a mobile gateway, and cloud-based storage, analytics, and clinical monitoring.Adapted from [218], licensed under CC BY 4.0

The reviewed work by Phan et al. provides a comprehensive perspective on wearable and IoMT-enabled biosensing systems, highlighting the convergence of multiparametric sensing, wireless communication, and cloud-based analytics [218]. Figure 15 serves as a concise visual abstraction of this paradigm, capturing how heterogeneous physiological signals acquired at the wearable level can be seamlessly integrated into an end-to-end IoMT infrastructure. This system-level representation complements the device-specific examples discussed throughout this subsection and underscores the importance of architectural integration for the practical deployment of multiparametric biosensing platforms.

In all these systems, multiparametric and networked operation raises additional system-level questions that go beyond the individual sensor node: how to fuse heterogeneous data streams, manage power consumption over long-term use, guarantee data security and privacy, and ensure interoperability with existing clinical information systems. These aspects naturally connect microwave hardware with ML and IoMT infrastructures, reinforcing the idea that advanced functionalities must be conceived as part of an integrated sensing–communication–analytics pipeline rather than as isolated hardware add-ons.

## Conclusions

This comprehensive review has provided a systematic and critical assessment of microwave technologies for biomedical diagnosis and therapy, highlighting their consolidation as versatile platforms that bridge sensing, imaging, and therapeutic intervention. By integrating electromagnetic fundamentals with advances in applicator engineering, biosensor architectures, clinical validation, and emerging intelligent frameworks, the manuscript positions microwave-based approaches as key enablers of non-invasive, cost-effective, and scalable solutions within precision medicine.

From a diagnostic perspective, microwave biosensors have demonstrated robust sensitivity across a broad range of biomedical targets. Label-free immunosensors for oncological biomarkers such as CEA and anti-p53 autoantibodies routinely achieve picogram-per-milliliter detection limits, approaching the performance of established biochemical assays while preserving non-ionizing operation and simplified workflows. Beyond oncology, metabolic monitoring platforms enable quantitative tracking of glucose and urea with MHz-level frequency shifts, and pathogen detection systems have reached clinically relevant thresholds, reinforcing the potential of microwave sensing for portable and POC diagnostics. Microwave imaging further contributes unique contrast mechanisms based on dielectric heterogeneity in soft tissues. Although its spatial resolution remains inferior to MRI and CT, its low cost, safety profile, and real-time capability support its role as a complementary modality rather than a replacement for conventional imaging techniques.

On the therapeutic side, microwave ablation has emerged as a mature and effective modality, particularly for tumors exceeding 3 cm and for anatomies where heat-sink effects limit RF ablation. Clinical evidence consistently reports improved local control and progression-free survival in selected scenarios, while preclinical developments in applicator geometries, phase-change materials, and image-guided workflows continue to enhance targeting accuracy and procedural safety. In parallel, microwave-based hyperthermia has evolved beyond standalone thermal treatment, demonstrating synergistic effects when combined with chemotherapy, radiotherapy, and immunotherapy, thereby reinforcing the role of microwaves as multifunctional therapeutic tools.

Likewise, the field is advancing toward convergence. Microfluidic integration allows precise sample handling at the microliter scale; wearable and implantable devices expand personalized, continuous monitoring; ML algorithms enhance diagnostic robustness and compensate for environmental variations; and IoMT-based multiparametric platforms create connected healthcare ecosystems. These innovations are progressively enabling theranostic systems to obtain platforms that unite detection, therapy, and monitoring within a single, adaptive framework.

Despite this substantial progress, several challenges must be addressed to enable widespread clinical translation. Dielectric measurements remain sensitive to physiological fluctuations and environmental perturbations, which can affect baseline stability and repeatability. Strategies such as temperature-compensated resonators, calibration-free interrogation schemes, and multi-frequency feature extraction are mitigating these limitations, but further validation under realistic clinical conditions is required. Reproducibility and standardization also remain critical concerns, as fabrication tolerances, substrate variability, and heterogeneous S-parameter reporting complicate cross-platform comparisons. Ongoing efforts toward unified benchmarking protocols, validated dielectric phantoms, and harmonized microfabrication practices represent essential steps toward inter-laboratory consistency.

For therapeutic applications, precise spatial control of energy deposition continues to be challenged by tissue perfusion and anisotropy; however, advances in phased-array steering, adaptive power modulation, and integrated temperature monitoring are steadily improving treatment accuracy and safety. In biosensing, biochemical selectivity in complex biofluids remains a limiting factor for purely dielectric approaches, yet hybrid biochemical–dielectric architectures and AI-assisted feature extraction are demonstrating promising gains in specificity. Finally, translational and regulatory barriers are being progressively addressed through long-term stability studies, miniaturized readout electronics, and alignment with emerging clinical electromagnetic-exposure standards.

## Data Availability

No datasets were generated or analysed during the current study.

## Declarations

### Clinical trial registration details

This study does not involve a clinical trial. Clinical trial registration: not applicable.
